# PlantDefender: a unified deep learning approach for maize disease and pest detection

**DOI:** 10.3389/fpls.2026.1960161

**Published:** 2026-10-12

**Authors:** Saleh Albahli, Marriam Nawaz, Tahira Nazir

**Affiliations:** 1 Department of Information Technology, College of Computer, Qassim University, Buraydah, Saudi Arabia; 2 Department of Software Engineering, University of Engineering and Technology-Taxila, Taxilia, Pakistan; 3 Faculty of Computing, Riphah International University, Islamabad, Pakistan

**Keywords:** deep learning, faster-RCNN, InceptionResNet-V2, maize diseases, pests, SWISH

## Abstract

**Introduction:**

Maize crops face severe threats from various diseases and pests, significantly affecting crop quality and yield. Prompt and accurate diagnosis is essential to minimize agricultural losses. Existing models have demonstrated limitations in handling complex backgrounds, inter- and intra-class variations, and inefficient feature extraction, which complicate the recognition of maize diseases and pests.

**Methods:**

This study proposes a unified deep learning model, termed PlantDefender, for the simultaneous recognition of maize diseases and pests. PlantDefender employs an improved Faster R-CNN architecture with a refined InceptionResNet-V2 as its backbone. The conventional InceptionResNet-V2 is modified by replacing the ReLU activation function with Swish to enhance feature extraction. The extracted features are subsequently processed through the Region Proposal Network (RPN), ROI Align, regression, and classification modules for accurate detection and recognition. The model is evaluated using the IP102 dataset for maize pest recognition and the CD&S dataset for maize leaf disease recognition.

**Results:**

PlantDefender achieves an accuracy of 78.67% on the IP102 dataset and 98.44% on the CD&S dataset. The results demonstrate effective localization and classification performance across both maize pest and disease recognition tasks.

**Discussion:**

The experimental results indicate that the proposed PlantDefender framework can effectively address the challenges associated with the recognition of maize diseases and pests. By integrating the refined InceptionResNet-V2 backbone with Faster R-CNN, the proposed unified model provides a promising approach for automated and accurate maize crop protection.

## 1 Introduction

Plants and crops are the foundation of farming, which endures the economies of nations globally. In many developed and underdeveloped countries, agriculture is the major area providing occupation to several people and establishing the root of their economies (Chiocchio et al., 2021; Yang et al., 2026). Various crops, including rice, wheat, maize, and soybeans, not only act as a staple food for a substantial portion of the people around the globe but also serve as a major export commodity that drives financial development (Campos et al., 2020). In advanced nations, modern farming practices using new technological advancements have resulted in a huge production and export of high-quality crops (Singh et al., 2026), which contribute incredibly to the state’s GDP (Kumar and Verma, 2021). Further, the field of farming has a close relationship to other trades, for example, the field of food processing, textiles, and biofuels, developing a multiplier influence that stimulates monetary evolution. So, based on the above-mentioned details, it can be said that the health and output of plants are directly linked to food security, revenues, and economic stability globally (Pandey and Pandey, 2023). However, there exist various factors directly impacting the crop yield that can extremely disturb the quantity and value of agrarian harvest (Furtak and Wolińska, 2023). These factors include environmental conditions, for example, temperature, rainfall, and soil quality. Then, unexpected weather conditions like droughts, floods, and variations in temperature can cause crop damage and result in reduced harvests and monetary losses for agriculturalists (Çakmakçı et al., 2023). Furthermore, human activities like deforestation, contamination, and unmanageable agronomic practices also result in the dilapidation of soil quality and disturb ecosystems, affecting crop growth (Roy et al., 2023). In addition, various pathogens and pests pose a substantial danger to the health of crops as they have the potential to propagate fast, causing extensive damage and resulting in considerable financial damage and food shortages (Bouri et al., 2023). The impact of reduced plant yield is extensive, which not only affects individual farmers but also national food security and global food supply chains. To ensure consistent and high crop yields, it is mandatory to manage these factors through sustainable agricultural practices, technological innovations, and effective control measures.

Among all the listed factors above, plant diseases and pests are the most challenging ones disturbing both the crop yield and value (He et al., 2023). Diseases caused by fungi, bacteria, viruses, and other pathogens can lead to severe crop losses. Plant diseases often appeared as wilting, yellowing, and spotting, and stopped progress in plants and impaired their capability to grow healthy yields (Tang et al., 2023). While pests comprising insects, mites, and nematodes breed on plant tissues, sap, and roots, this results in physical damage to plants and sometimes spreads viruses. These pests have the capability to infiltrate large ranges quickly and cause extensive crop damage. Hence, the economic impact of plant diseases and pests is considerable, which causes decreased agronomic output, increased costs for pest and disease management, and reduced marketability of crops (Nayagam et al., 2023). To alleviate the effect of such biotic stressors and guarantee the permanency of universal food production, it is the need of the hour to design effective management strategies like integrated pest management, resilient crop varieties, and sustainable farming practices. To diagnose plant diseases and pests, initially, manual techniques were adopted by the agriculturalists. To perform this, farmers and agricultural workers usually physically inspect crops for signs of diseases and pests, i.e., stained leaves, spots, or visible insects (Khan et al., 2023). Then, based on the observations, various pesticides and other control measures were adopted. These approaches also involve crop rotation, intercropping, and the use of resistant plant varieties. While such methods have been effective to some extent, they come with several limitations. Manual inspection is time-consuming and labor-intensive, which demands a skilled workforce. These approaches are also open to human error due to the reason that the early signs of disease or pest infestation can be easily missed which eventually cause delayed intervention and more severe outbreaks. Also, the huge dependency on chemical pesticides has environmental and health consequences comprising soil degradation, water contamination, and pesticide resistance to pests. These challenges highlighted the need for more efficient and accurate approaches to plant disease and pest management (Khang et al., 2023).

To tackle the issues sustained in manual practices, the research community has designed various automated systems by employing current technological advancements. At first, methods comprising conventional machine learning (ML) approaches were evaluated to formulate automated solutions for the recognition of plant diseases and pests (Venkatasaichandrakanth and Iyapparaja, 2024). These techniques have the ability to analyze datasets by identifying patterns and their relationships that are hard to detect through manual surveillance. To perform this, researchers use various ML approaches like image recognition and pattern analysis, supporting precise and timely identification of disease signs and pest invasions. The ML approaches have minimized the reliance on human effort and optimized efficiency, resulting in the correctness of the recognition process; however, these methods come with various limitations. These methods rely on the availability of labeled data to tune the model, which is difficult and costly to acquire. Secondly, these solutions are not proficient in tackling sample alterations like variations in lighting, background, and plant conditions, which makes them unreliable in practical solutions. Moreover, ML approaches are not effective in learning complicated patterns of disease development and pest behavior, which limits their usefulness in diverse farming environments (Morchid et al., 2024). To overcome the limitations of traditional ML techniques, scientists have adopted deep learning (DL) approaches. Various DL techniques like convolutional neural networks (CNNs) have depicted notable capabilities in executing numerous image recognition tasks, which makes them well-suited for plant disease and pest detection (Christakakis et al., 2024). DL solutions are more capable of learning an informative set of sample characteristics from raw data and minimizing the constraints of manual feature computation (Sunil and Jaidhar, 2021; Sunil and Jaidhar, 2024). Further, these methods are more robust in dealing with complex patterns and representations, which permits them to improve the accuracy and effectiveness of disease and pest identification. Further, these methods are more proficient in dealing with the variability in data, for example, transformations in lighting, background, and plant species, which makes them more adaptable to real-world conditions. Also, these models can assimilate multiple data sources like images, weather data, and soil information to deliver comprehensive and intelligent predictions.

Despite the mentioned advantages of DL approaches in recognizing plant pests and diseases, there still exist various challenges that need further enhancements (Sunil et al., 2023; Ali et al., 2024). One important issue is the need for large, annotated data samples to tune effective DL models, which is a time-consuming activity, especially in diverse agricultural environments. Additionally, DL models are computationally intensive and require high-performance hardware for training and inference, which is usually not accessible to all farmers or researchers. Another limitation is the generalizability of DL models, as these methods perform well under controlled conditions; however, they struggle with variability in real-world scenarios like different illumination settings, plant growth phases, and environmental factors (Demilie, 2024). Furthermore, DL models are black boxes in nature, which makes it difficult to interpret their decisions and understand the underlying mechanisms of disease and pest identification. One of the most notable limitations is that a few works in history have been designed that present a unified model capable of detecting both plant diseases and pests through a single system (Sajitha et al., 2024). Currently, most DL models are specialized for either disease detection or pest identification, demanding multiple models and increasing the complexity of implementation and maintenance. Addressing these limitations is crucial for the widespread adoption and effectiveness of DL approaches in agricultural applications. In our research, we have designed an effective unified model capable of detecting both plant diseases and pests, specifically focusing on maize (corn) plants. Maize is a high-ranked cereal crop worldwide, considered a staple food for humans and a key component in preparing animal feed. This crop is also recognized as a major raw material used in the preparation of numerous manufacturing products, for example, biofuels and bioplastics. Due to its immense importance, many countries around the globe cultivate it, among which the United States, China, Brazil, India, and Saudi Arabia are ranked as top contributors (Wu and Guclu, 2013). The global importance of maize is emphasized by its versatility and high nutritional value, which highlight it as a crucial element of food security and economic stability in many regions. Maize is used in a variety of products ranging from corn syrup and cornmeal to ethanol and biodegradable plastics. The above-listed factors highlight its business value. However, the yield of maize is often disturbed by various plant diseases and pests, which can severely impact both the quantity and quality of the crop (Erenstein et al., 2022).

In this study, we develop PlantDefender, a unified deep learning framework for the localization and classification of both diseases and insect pests affecting maize crops. Here, “unified” refers to the use of a common detection architecture and feature-extraction strategy for both maize pest and disease recognition rather than joint training of the two datasets. Due to the different annotation structures, target characteristics, and visual domains of IP102 and CD&S, the two datasets were trained and evaluated independently using the same PlantDefender architecture and experimental protocol. This design enables the applicability of the proposed framework to both maize pest and disease detection while maintaining dataset-specific training and evaluation. The framework is built upon the Faster R-CNN detection architecture and incorporates a refined InceptionResNet-V2 feature extractor in which the conventional ReLU activation is replaced with Swish. Although these individual components have been established in previous deep learning research, their task-specific integration in PlantDefender is designed to address the distinct visual characteristics of maize diseases and insect pests within a common detection pipeline. The smoother nonlinear response of Swish facilitates continuous gradient propagation and enables the backbone to learn discriminative representations from complex agricultural images, while the region-based Faster R-CNN detector subsequently performs region proposal generation, localization, and classification. Consequently, PlantDefender provides a common framework for detecting relatively stationary disease symptoms on maize leaves as well as visually diverse pest instances, demonstrating that the same detection architecture can be applied to both disease and pest recognition without requiring separately designed architectures for the two tasks. The effectiveness of the framework is evaluated using two complementary datasets: the IP102 dataset, from which 13 maize-related pest categories are selected, and the CD&S dataset containing three major maize diseases. The main contributions of this study are summarized as follows:

- Unified Detection Framework: The study proposes a common DL framework capable of addressing both maize plant disease and pest detection using the same refined feature-extraction and region-based detection architecture, thereby providing a unified methodological solution while allowing dataset-specific training and evaluation.
- Task-specific feature-extraction refinement: We refine the InceptionResNet-V2 feature extractor by replacing ReLU with Swish and integrate the refined backbone with Faster R-CNN to enhance nonlinear feature representation for visually complex maize disease and pest images.
- Integrated localization and classification: The proposed framework jointly exploits region proposal, bounding-box regression, and classification mechanisms to identify the location and category of disease symptoms and pest instances rather than performing classification alone.
- Cross-scenario experimental validation: We evaluate PlantDefender on two complementary maize datasets, including 13 maize-related pest categories from IP102 and three disease categories from CD&S, allowing its performance to be assessed across substantially different visual characteristics and detection scenarios.
- Practical relevance to intelligent phytoprotection: By providing a unified automated mechanism for maize biotic-stress detection, PlantDefender has the potential to support timely crop-health assessment and precision agricultural management.

The latter paper is distributed into the following parts: Section 2 contains literature work, while Section 3 explains the detailed methodology. We have discussed the datasets and attained performance over them in Section 4. While Section 5 concludes and explains the future directions.

## 2 Literature review

This part discussed the works from history presented for the recognition of maize plant leaf diseases and pest recognition.

First, we have elaborated on the approaches proposed by researchers for the classification of maize plant leaf diseases. Halloran et al (O’Halloran et al., 2024). proposed a DL framework for accomplishing the task of maize plant leaf diseases. For this, the approach employed 6 DL frameworks with one as the basic CNN model comprising various convolution layers along with the classification module, while the rest of the 5 frameworks were based on pre-trained DL networks. For tuning all 6 models, the approach utilized a custom dataset and reported the highest classification results of 99.99% with the B0-based EfficientNet-V2 approach. This work shows an effective classification score; however, the model needs testing on a larger and standard dataset to prove its efficacy. A huge amount of maize plants are cultivated worldwide, but diseases are the main threat to maize production, affecting both the quality and quantity of the produce. Leaves can be utilized to recognize these diseases easily. To address this issue, the work (Jasrotia et al., 2023) proposed an automated solution for diagnosing maize plant ailments. A customized CNN-based Maize Plant Disease Identification model was developed, incorporating preprocessing approaches, for example, Contrast Limiting Adaptive Histogram Equalization (CLAHE) on each RGB channel, log transformation, and RGB to HSV conversion. These trained models were compared to various DL and ML techniques trained without applying any pre-processing phase. The work was tested on the PlantVillage Maize Crop Dataset and achieved a maximum accuracy of 96.76%. Even though the work improved the recognition ability of maize plant leaf diseases, the approach needs evaluation of a more diverse dataset with altered weather conditions to prove its robustness. Timely detection of plant abnormalities is crucial for protecting crop output, especially in areas facing food insecurity. Northern Leaf Blight (NLB) is a major threat to maize production, typically taking 14–21 days to show visible signs. For this, Maginga et al. (2024) suggested an IoT sensor-based approach to detect NLB within a minimum period. By employing CNN and LSTM frameworks, the study analyzed nonvisual data from Total Volatile Organic Compounds (VOCs) and ultrasound emissions of maize plants. This hybrid technique secured an F1 of 0.96 along with 99.98% accuracy in anomaly detection. The approach presents an effective framework for maize plant leaf disease recognition; however, with a huge computing cost. The approach discussed in (Liu et al., 2024) introduces a residual-oriented multi-scale network (MResNet) for the recognition of numerous viruses from the maize plant leaves. The architecture of the MResNet approach comprises 2 residual units with varying scales capable of locating the abnormalities of maize leaf samples at altering scales. Further, a hybrid key points weight optimization approach was included to boost and merge the information captured by both units. The approach was tested on a maize leaf disease dataset, achieving an accuracy of 97.45%; however, it lacks to exactly locate the location of disease regions. Khan et al. (2024) employed the idea of transfer learning to recognize the various abnormalities found in the maize plant visual samples. For this, the approach has taken 4 well-known DL approaches and tuned them in an end-to-end manner to accomplish the classification task. The work utilized the samples from various repositories to develop a custom dataset and reported the highest results with the ResNet50 approach with an accuracy value of 87.51%. The work performs well for maize plant leaf disease recognition, however, the results require more improvements.

Khan et al (Khan et al., 2023). suggested an approach by taking the concept of using the object locator approach to recognize the infected maize plant leaf samples. This work allowed for both spot recognition and classification labels of infected regions. The dataset, which included 3 specific maize diseases, was gathered under altered weather conditions to tune and evaluate the model performance. The work has analyzed various variants of the YOLO model, ranging from v3 to v8, and reported the highest recognition results with the YOLOV8n approach, with a score of 99.04%. The approach performs well; however, the model lacks to locate the small infected regions. Nan et al. (2023) suggested an approach where they employed a multisensor coordinated RGB-D camera to categorize the ailments of maize plants, overwhelming encounters of conventional techniques in complicated real-world scenarios. The approach tested 4 DL frameworks named ResNet50, MobileNetV2, VGG16, and EfficientNet-B3 for this purpose. Assessments between pre-segmentation and post-segmentation methods showed pre-segmentation approaches slightly outperforming, but overrating performance because of a cluttered background. Post-segmentation models focusing on leaf disease features provided practical results with shorter prediction times. ResNet50 and MobileNetV2 excelled, with MobileNetV2 particularly notable for compactness and speed; however, the approach needs evaluation on a large data sample. Masood et al. (2023) proposed an approach by employing an object detection approach for recognizing various corn leaf abnormalities. For this, the approach has utilized ResNet50 with an attention mechanism as the base network of the Faster-RCNN approach. The work attained an accuracy value of 97.89%; however, unable to tackle intense light variations. Patel et al. (2023) employed a conventional ML approach for recognizing corn plant leaf ailments. By utilizing texture examination with Gray-Level Co-occurrence Matrix (GLCM) and Gabor keypoints computation on the PlantVillage data sample, the work used Decision Tree, Gradient Boosting (GB), SVM, and KNN to categorize normal and infected samples. The approach has reported the highest classification results of 95.05% with the GB predictor. The approach presents an efficient solution to corn leaf diseases; however, unable to tackle the complex environmental conditions. Siam et al. (2024) employed various DL approaches in an end-to-end manner with a transfer learning concept for the recognition of maize plant viruses. For this, the work has taken into account numerous reputed DL approaches like VGG16, ResNet50, and MobileNetV2, etc., to further focus on the targeted regions, the work has integrated the attention strategy into the DL approaches. This work has attained the highest classification score of 94.76% with the MobileNetV2 approach, however, unable to exactly spot the areas of infected portions. The work in (Li et al., 2023) suggested Sim-ConvNeXt, a CNN approach with a parameter-free SimAM attention unit, boosting visual information capturing capability and categorization accuracy for maize illnesses. Transfer learning was used to speed up model tuning and improve outcomes. Using a dataset of 17,670 images expanded through data augmentation, the model achieved 95.2% accuracy, outperforming the ConvNeXt and Swin Transformer models by 1.2% and 1.5%, respectively, however, with extensive computing burden.

Next, we have discussed the works presented in the literature for recognizing various types of maize pests. Recent advances have also explored the integration of attention mechanisms with deep convolutional architectures for plant disease recognition. The study in (Khan et al., 2025) proposed a Dense-Inception-based architecture incorporating attention modules for plant disease classification, demonstrating the potential of combining multi-scale feature extraction with attention mechanisms to enhance discriminative representation learning. Such approaches are particularly useful for distinguishing disease-related visual patterns under varying image conditions. However, the study primarily focuses on plant disease classification.

Saadati et al (Tao et al., 2022). employed a DL approach for recognizing worms and damage caused by them on the maize plant. For this, the work has taken 4 well-known DL methods called AlexNet, DenseNet, EfficientNet, and GoogleNet. These approaches were tuned on a data sample comprising 4500 images and trained in an end-to-end strategy using pre-trained weights. The approach reported the highest results for the EfficientNet model, with an accuracy of 99.68%. The work has performed well for maize worms; however, the techniques are evaluated over a small dataset and need evaluation on a larger and standard visual dataset to prove the effectiveness. In another work discussed in (Haq et al., 2023), the authors investigated 2 DL models under the TensorFlow framework to recognize fall armyworm pests from maize plant leaves, utilizing a data sample with 7,000 images collected from Shandong University. The approach considered 2 DL models called VGG-16 and Cov2D, in comparison to various DL approaches like AlexNet, ResNet, and Yolo variants. VGG-16 and Cov2D showed superior accuracy and ease of training, with VGG-16 particularly suitable for detecting new pests. The approach performs well; however, unable to spot the pests’ positions on the plants and lacks generalization ability. Plant pests often decrease crop value and profit. The extensive resemblance and quick movement of pests complicate the prompt identification and categorization of pests. To overcome this, the work (Yang et al., 2023) developed Maize-YOLO, an efficient and high-precision, real-time maize pest recognition approach by employing YOLOv7, enhanced with CSPResNeXt-50 and VoVGSCSP units. These additions improved detection accuracy and speed while reducing computational demands. The approach was tested by employing the maize pest samples from the IP102 data sample, which comprised 13 pest classes harmful to maize. This work achieved performance values of 76.3% mAP and 77.3% recall; however, the results need further enhancements. Kirongo et al. (2024) suggested an approach to classify various corn pests from visual samples. For this, the work has designed a CNN approach comprising various convolution layers with a classification module. The approach has collected a small data sample of maize pests for model tuning and testing. The work shows effective classification behavior; however, it must be evaluated on a complex and standard dataset for model verification. Further, the approach lacks exactly locating the pest position. Appiah et al. (2024) suggested a DL approach to recognize the pests from 3 types of plants, including maize. For this, the approach has employed the EfficientNetB3 technique to extract the visual characteristics of samples. The extracted information was later used in a CNN classifier to accomplish the classification task. The approach performs well in pest classification, however, the approach was evaluated with self-collected images and needs testing on a standard dataset. YOLO-based detectors have also been increasingly investigated for agricultural object detection. The study in (Raza et al., 2025) benchmarked different YOLO models for crop growth and weed detection in cotton fields, highlighting the applicability of single-stage detection architectures to agricultural environments with complex field conditions. The findings further demonstrate the importance of selecting an appropriate detection architecture according to the characteristics of agricultural targets and environments.

Some existing works attempted to propose approaches that perform the recognition of both maize plant leaf diseases and pests. One such framework was presented in (Resti et al., 2023a), where the authors employed the conventional ML approach for classifying corn pests and viruses, as these posed a dangerous threat to worldwide food safety and sustainable farming. The work utilized the random forest (RF) approach, a powerful tree-based classification framework, which benefits from bootstrap aggregation to boost results. By combining forecasts from numerous decision trees, this approach accomplished the categorization task. The approach performs well for maize plant viruses and pests classification; however, unable to tackle distorted images. Maize is heavily impacted by pests and viruses, which substantially affect its quality and harvest. To overcome this, a DL approach was suggested in (Xu et al., 2023) to automatically classify both maize plant diseases and pests by using ResNet50. By assimilating added channels and filtering convolutional processes, the approach gathered a dense set of visual information from the given samples and alleviated framework degradation. Experimental validation demonstrated a recognition accuracy of 96.02%; however, the approach is unable to locate the exact positions of pests and infected plant areas. Maize, a critical worldwide crop, faces substantial encounters with ailments and pests. For this, an automated framework was proposed by Liu et al. in (Liu et al., 2024) called the LFMNet (Maize Leaf Disease and Pest Identification Network) framework, by including modules like Localized Multi-Scale Inverted Residual Convolutional Block (LMSB) for capturing the visual aspects of input images, Feature Localization Bottleneck (FLB) for improved illness and pest description, and Multi-Hop Local-Feature Fusion Architecture (MLFFA) for better keypoints estimation and amalgamation. Tested on 19,451 samples, LFMNet achieved a high accuracy of 95.68%, however, with an extensive computing burden. Kang et al. (2023) suggested an enhanced CenterNet approach by merging an attention mechanism with ResNet50 to alleviate multifaceted contextual intrusion and improve visual information extraction from the maize plant visuals containing various diseases and pests. A multi-measure keypoint combination unit merging local and global aspects of samples was used to improve the results. The approach was tested using a self-collected repository of samples and reported 85.13% average detection accuracy. The work performs well for the designated task, however, needs further result improvements. The work presented in (Resti et al., 2023b) explored corn plant illness and pest organization employing digital samples boosted by fuzzy discretization. Fuzzy discretization permitted for overlying breaks in group definitions, and compliant unclear data. Diverse membership method groupings were verified to enhance cataloging results, and evaluated through Monte Carlo resampling. The best model, identified by high F-score and Kappa values, assimilated digital image preprocessing and classification methods, however, the approach accomplished image-level classification and was unable to locate the targeted areas.

Our detailed analysis of existing approaches for maize plant leaf disease and pest classification highlights separate and unified methodologies designed for each category. While these methods demonstrate individual promise, their integration and overall efficiency when applied together are often lacking. Current approaches tend to prioritize either disease or pest classification, resulting in fragmented solutions that underutilize shared insights and compromise overall detection accuracy. This underscores the necessity for a more effective unified approach capable of simultaneously addressing both maize plant diseases and pests.

## 3 Methodology

In this work, we have developed a powerful DL approach called PlantDefender to address the challenges of detecting plant diseases and pests in maize crops simultaneously. The technique employs an enhanced Faster-RCNN (Albahli et al., 2021) architecture, which is famous for its exceptional capability in object detection by effectively localizing and classifying objects within images. For feature extraction, we employed a refined InceptionResNetv2 (Nawaz et al., 2024) architecture as the base network of the Faster-RCNN model by substituting the traditional ReLU activation function with Swish. The inclusion of Swish enhances the flow of gradients in the network and boosts overall performance by tackling issues like vanishing gradients through enhanced feature computation. The learned information is then processed by the PlantDefender model by effectively utilizing its region proposal network (RPN) to identify potential regions of interest in the images. Finally, a classification network accurately categorizes these regions into specific disease types or pest infestations. We have tuned and validated the proposed work on 2 challenging data samples named IP102 and CD&S to confirm the effectiveness of our approach for pest recognition and maize plant diseases, respectively. The entire flow of the PlantDefender approach is given in **Figure 1**.

**Figure 1:**
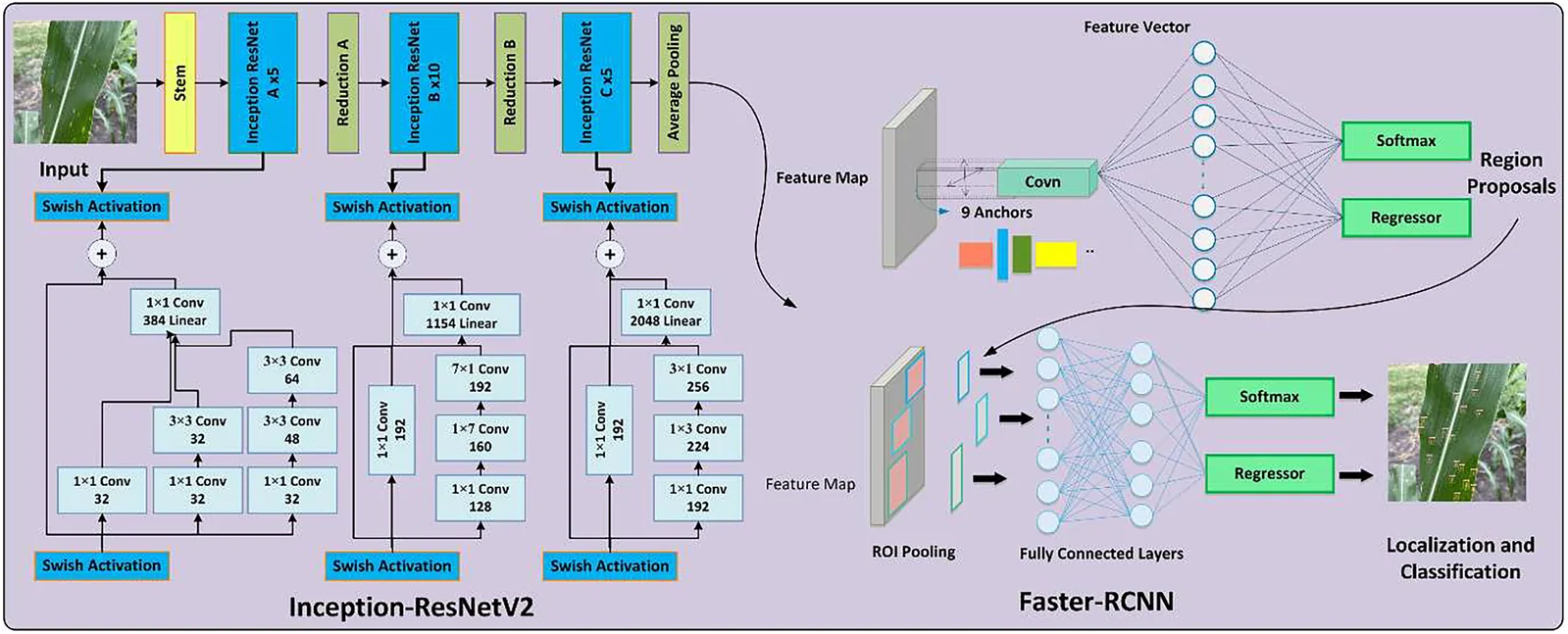
Workflow diagram of the PlantDefender approach.

### 3.1 Faster RCNN

Faster R-CNN is a cutting-edge object detection model recognized for its efficiency and accuracy in identifying and localizing objects within images. It improves upon earlier models like RCNN and Fast-RCNN by integrating an RPN module that shares convolutional layers with the detection network. Such a model description significantly reduces computational costs, enhances processing speed, and ensures that Faster-RCNN processes images faster and more accurately. The efficiency of the model stems from the RPN module, which generates high-quality region proposals quickly in comparison to the computationally expensive selective search used in RCNN and Fast-RCNN. The Faster-RCNN approach utilized its shared convolutional layers for feature map enhancement to improve the target recognition results, particularly for tiny or overlapped objects. Such a model description makes it a preferred choice for problems requiring comprehensive and accurate object recognition.

In our case of proposing a unified solution for maize plant disease and pest identification, the choice of Faster R-CNN offers considerable advantages. For example, the capability of the model to handle complex and cluttered backgrounds makes it suitable for real-world agricultural environments. Further, its flexible architecture allows it to adapt to various scenarios through the employment of refined feature maps, which assist us in accurate disease and pest detection. Such model architecture is mandatory for early and precise identification and critical for effective crop management and yield protection. Contrary to various other object recognition techniques, the Faster-RCNN model provides a balanced solution, as different variants of YOLO boost network efficiency by compromising accuracy with smaller or overlapping objects. Further, a 1-stage detector like RetinaNet addresses the class imbalance problem; however this technique is more complex and slower. While models like CornerNet and CenterNet provide precise localization, however struggle with very small objects. In comparison, the Faster-RCNN model offers an accurate solution with superior accuracy and robustness across several image transformations with balanced model efficiency.

### 3.2 Improved faster RCNN

The conventional Faster-RCNN approach offers effective object recognition performance; however, it still faces some challenges due to the employment of backbone networks like VGG16 and ResNet101.

These base networks cannot fully capture the relevant sample details, especially in complex tasks like detecting plant diseases and pests with varying environmental settings. The VGG16 architecture is computationally expensive and less efficient in learning complex visual aspects of images due to its uniform architecture and lack of residual links. Whereas, ResNet101 involves residual connections that assist the dense networks to tune well, however, it still lacks in accurately extracting diverse features from high-resolution images. |Such issues result in the degradation of overall model performance and accuracy to effectively recognizing the target spots (i.e., maize diseases and pests). Therefore, there is a need to adopt a more robust and reliable feature extractor to overcome the above-listed issues and ensure higher detection accuracy in complex agricultural environments.

#### 3.2.1 Refined inceptionResNet-V2

InceptionResNet-V2 is an influential NN model that offers effective results by combining the strengths of Inception modules and residual connections from the ResNet approach. The combined architecture offers a more efficient and accurate feature set by using the multi-scale information learning power of the Inception modules and the improved recall ability of the residual connections. The InceptionResNet-V2 is competent to capture the image details at different scales, which makes it well-suited for tasks requiring detailed feature sets like in our case of identifying diseases and pests in maize crops. The proficiency of InceptionResNet-V2 to process samples with numerous filter sizes and depths concurrently permits it to learn complex sample details more effectively. Despite the mentioned benefits, the InceptionResNet-V2 approach comes with some limitations. The employment of the ReLU activation function among its convolutional layers leads to issues like vanishing gradients, which hinder the model from learning complex patterns in deeper layers and result in suboptimal feature computation and classification outcomes. Even though the InceptionResNet-V2 is more efficient in comparison to traditional networks like VGG16, it still needs enhancements due to its inability to fully utilize the potential of smoother activation functions that could further enhance gradient flow and overall model performance. To address the mentioned problems, we have proposed a refined InceptionResNet-V2 by substituting the traditionalQ12 ReLU activation function with Swish, which is defined in Equation 1:

(1) $$Swish\left(x\right)=x/\left(1+e^{-x}\right)$$

The Swish method suggests a smoother and more flexible activation in comparison to ReLU. This substitution assists the model in overcoming the vanishing gradient problem and improves its ability to effectively learn complex sample details. Swish permits a better flow of gradients through the network, which boosts the feature computation ability of the model. So, the inclusion of Swish in the convolutional layers of the model assists our refined InceptionResNet-V2 to better handle the complex sample variations and varied features present in maize plant images. Such a better information-capturing ability of the feature extractor ultimately enhances the accuracy and reliability of our disease and pest detection model. The visual depiction of the refined InceptionResNet-V2 approach is provided in **Figure 2**, from which it can be seen that the model comprises 6 modules to compute the visual information from the maize samples. For our work, each module of the refined InceptionResNet-V2 architecture plays a crucial role in learning relevant feature sets and assists in boosting the recognition ability of the PlantDefender approach. Block A, known as the stem, starts the feature learning process by preprocessing maize leaf images. This part comprises convolutional layers along with pooling operations to minimize spatial dimensions and increase the depth to effectively learn the basic characteristics of visuals. Module B comprises Inception modules and is competent in concurrently processing features through multiple convolutional pathways of varying window sizes. Block B allows the network to capture both local and global context within the maize leaf images, which assists the network in learning differences in different types of diseases and pests. Module C contains residual connections, which are required to maintain gradient flow during training. These links propagate the information from the previous to the next layers and increase the recall ability of the network by enabling it to learn more robust representations of complex maize leaf patterns. Module D contains auxiliary classifiers or intermediate layers that aid in regularization and feature improvement. This module enhances the generalization of the feature extractor for different maize pests and diseases and enhances overall prediction accuracy. Module E is focused on compressing the learned information by minimizing the feature space via methods like average pooling. This step results in a more compact representation of features for the final stage with reduced computational complexity. Finally, Module F integrates the extracted features. So, all the modules of the refined InceptionResNetv2 architecture with incorporated Swish function mutually stimulate to improve the capability of the PlantDefender approach to detect and classify maize diseases and pests. This effective approach aims to learn a more related set of image features, enhance model performance, and provide robust solutions for agricultural management and crop protection.

**Figure 2:**
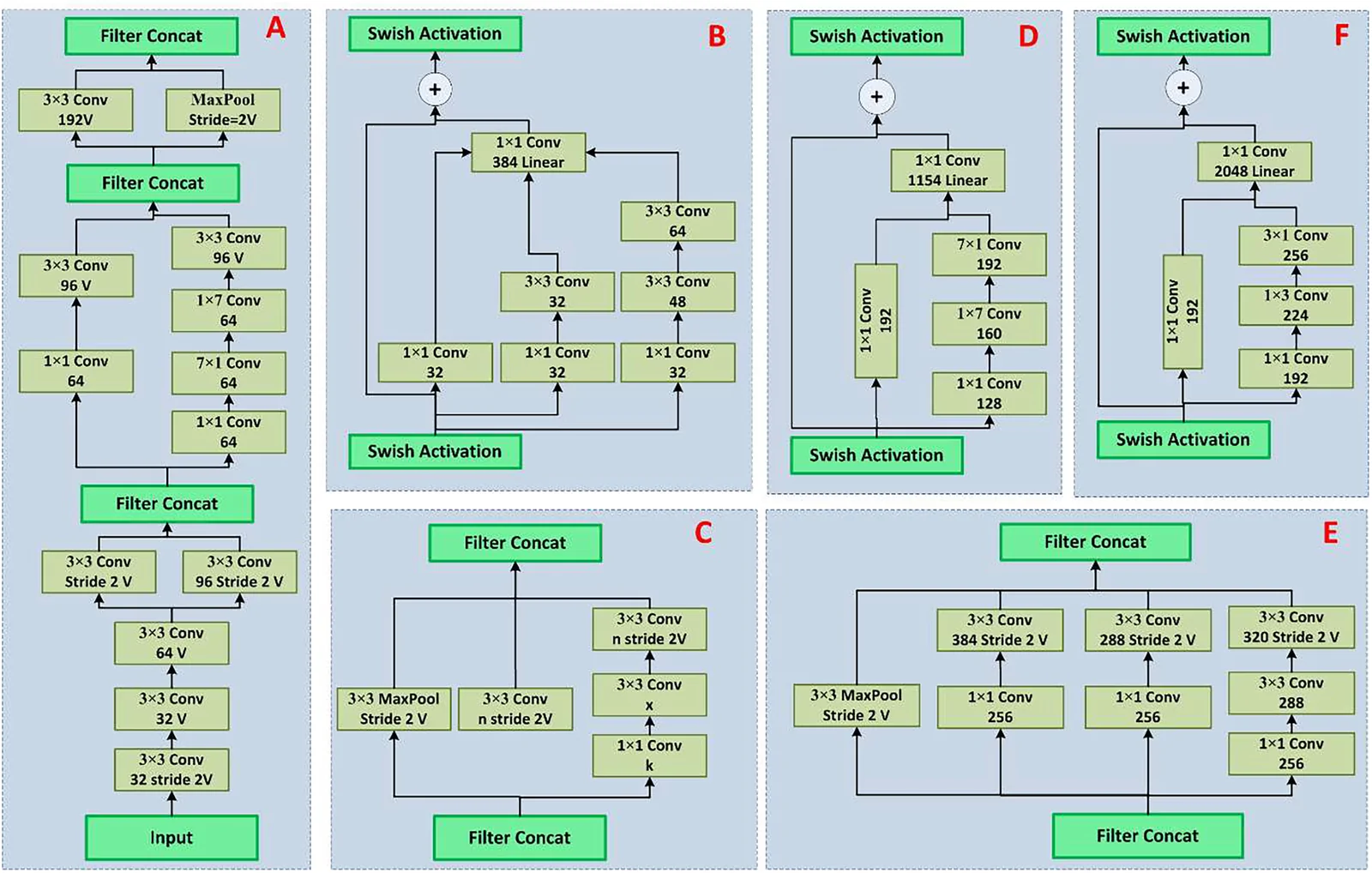
Representation of the refined InceptionResNet-V2 feature extractor: **(A)** Filter Concatenation block, **(B)** Swish-activated Inception-ResNet block, **(C)** Filter Concatenation with stride-2 downsampling, **(D)** Swish-activated reduction block, **(E)** Filter Concatenation with stride-2 downsampling, and **(F)** Swish-activated feature extraction block.

### 3.3 RPN

Next, the computed information from the refined InceptionResNet-V2 model is passed to the RPN unit, which processes the features to generate anchor boxes that serve as potential regions of interest (ROIs) where diseases or pests may be present. The RPN module generates the anchors at varying scales and aspect ratios to comprehensively cover the features extracted by refined InceptionResNet-V2. This module employs convolutional operations to evaluate the likelihood of each anchor box containing relevant information for proposal refinements, which are later used in regression and classification modules. This method enables the model to effectively pinpoint areas on maize leaves that potentially exhibit disease symptoms or pest containment.

### 3.4 ROI align

Once possible ROIs are identified by the RPN modules, these are processed by the ROI Align layer of the PlantDefender approach. The major task of this layer is to tackle misalignment in ROIs and feature maps caused by quantization in the ROI pooling process. The ROI Align layer accomplishes precise spatial alignment by using the bilinear interpolation method to get the exact location on the feature maps through accurate estimation of the ROI’s spatial layout. Such alignments of ROIs are required to maintain spatial accuracy in object localization and improve the overall performance of disease and pest classification on maize leaves.

### 3.5 Regression layer

After the identification of ROIs by the RPN module and their alignment by the ROI Align layer, the next phase in our problem involves the Regression network. The major task of this unit is to estimate the coordinates of the bounding box for each recognized ROI. It employs computed regression offsets to adjust the initial bounding box coordinates to better fit the precise boundaries of the detected maize plant diseases and pests. This unit ensures that the ROIs are associated accurately with the actual objects in the image to improve the localization of our model. The regression module of the PlantDefender approach enhances the target recognition ability of the network, which in turn improves the overall performance of the approach.

### 3.6 Classification module

The classification unit is the final module of the PlantDefender approach, which assigns a specific class label to each ROI based on the extracted features. It performs the categorization of numerous types of maize plant diseases and pests detected within the ROIs. The Classification network uses the features extracted by the ROI Align layer to make precise predictions and ensure that each identified region is categorized correctly according to the type of disease or pest present. This unit contains fully connected layers with softmax activation, which permits the model to assign probabilities to each class.

### 3.7 Multi-task loss function

The proposed PlantDefender framework employs a multi-task loss function to jointly optimize classification and localization during training. As PlantDefender is based on the Faster R-CNN model, the overall loss is computed as the sum of classification loss and bounding-box regression loss, which enables simultaneous object categorization and localization. The total loss function is defined in Equation 2.

(2) $$L\;=\;L_{cls}\;+\;\lambda L_{reg}$$

Where Classification loss is denoted as *L_cls_* is defined in Equation 3.

(3) $$L_{cls\left(p,u\right)}=-\log\left(p_{u}\right)$$

While regression loss is denoted as and is defined in Equation 4.

(4) $$L_{reg\left(t^{u},v\right)}=\sum_{i\in \left\{x,y,w,h\right\}}\;smooth_{L1}\left(t_{i}^{u}-\;v_{i}\right)$$

Thus, total loss is defined in Equation 5:

(5) $$L\left(p,u,t^{u},v\right)=L_{cls\left(p,u\right)}+\lambda \left[u\;\geq \;1\right]\;L_{reg\left(t^{u},v\right)}$$

Here, *L_cls_* is responsible for predicting the correct disease or pest category, while *L_reg_* used for precise localization. Here, *p* refers to the predicted class probability, and *u* represents the ground-truth class label, *t^u^* corresponds to the predicted bounding-box coordinates, and *v* indicates the ground-truth bounding-box coordinates. The parameter *λ* controls the balance between classification and localization losses and is set to 1 in the Faster R-CNN framework. Moreover, [*u* ≥ 1]. acts as an indicator function, ensuring that regression loss is calculated only for positive object proposals.

## 4 Results

In this part, the details of the used data samples, performance parameters, and results analysis are provided, which can be found in the later sub-sections.

### 4.1 Parameters

For testing the results of the PlantDefender approach, various quantitative parameters like mean Average Precision (mAP), accuracy, precision, F1-score, and recall are used in this approach. The accuracy of the PlantDefender approach is computed using Equation 6.

(6) $$Accuracy=\frac{TP+TN}{TP+FP+TN+FN}$$

Equation 7 presents the mAP score, where AP stands for average precision across all classes, and *t* is the test image. While *T* indicates the total sample set.

(7) $$mAP:=\sum_{i=1}^{T}AP(t_{i})/T$$

**Figure 3** shows the precision and recall visualization.

**Figure 3:**
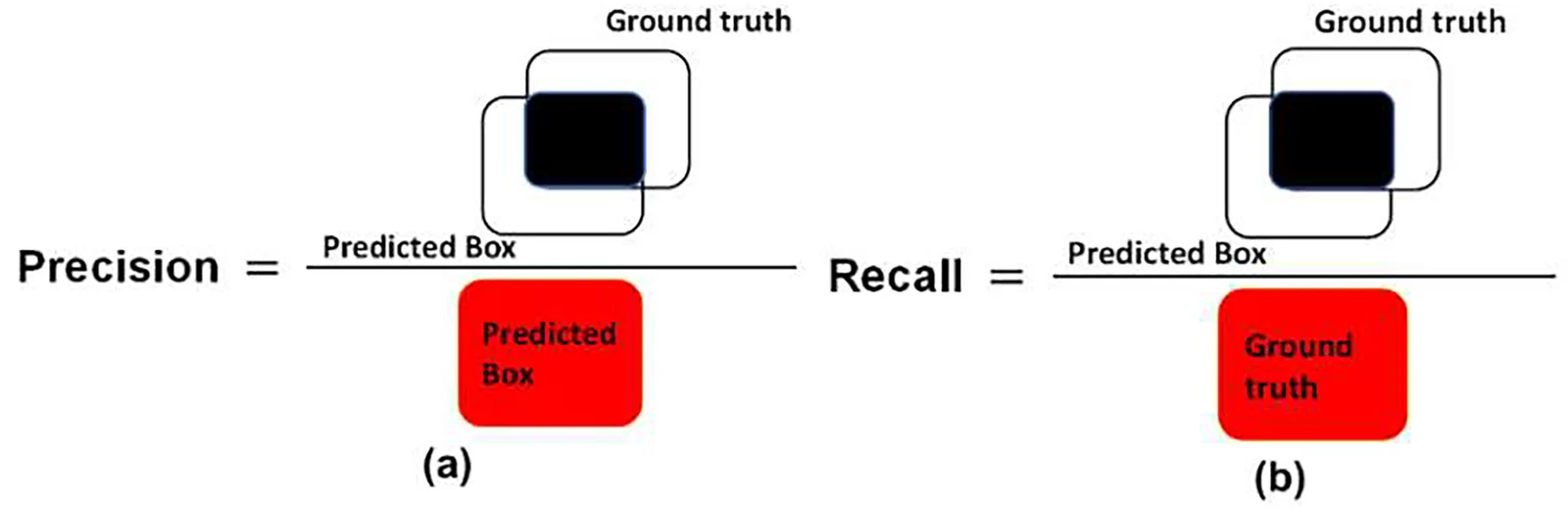
Representation of **(a)** precision and **(b)** recall.

### 4.2 Dataset preparation and experimental setup

To evaluate the maize plant disease and pest recognition capabilities of the PlantDefender approach, we selected two robust and publicly available datasets: IP102 (Wu et al., 2019) and CD&S (Ahmad et al., 2021). IP102 is a huge data sample designed for pest identification, containing 102 common crop pests. For our study, we focused specifically on the 13 pest species that affect maize due to their relevance to our research objectives. The corresponding images and their original annotations were retained for experimentation. No images were removed, duplicated, or synthetically generated during dataset preparation, and no modifications were applied to the original class labels or bounding-box annotations. This maize-specific subset provides a challenging evaluation scenario due to variations in pest appearance, scale, orientation, and background complexity. The CD&S dataset is sourced from Purdue University’s Agronomy Center for Research and Education and consists of 2112 high-resolution images capturing three prominent maize leaf diseases: Northern Leaf Blight (NLB), Northern Leaf Spot (NLS), and Gray Leaf Spot (GLS). The original disease categories and annotations were preserved throughout the experiments. These images were acquired under varying environmental conditions in field settings with diverse backgrounds and lighting conditions. This variability assists in ensuring the ability of a model to generalize across different scenarios encountered in agricultural practice. We chose these datasets for their diversity, relevance to maize crop health, and challenging characteristics, as these collectively enable comprehensive evaluation and validation of the PlantDefender approach. A few visuals from both data samples are shown in **Figure 4**.

**Figure 4:**
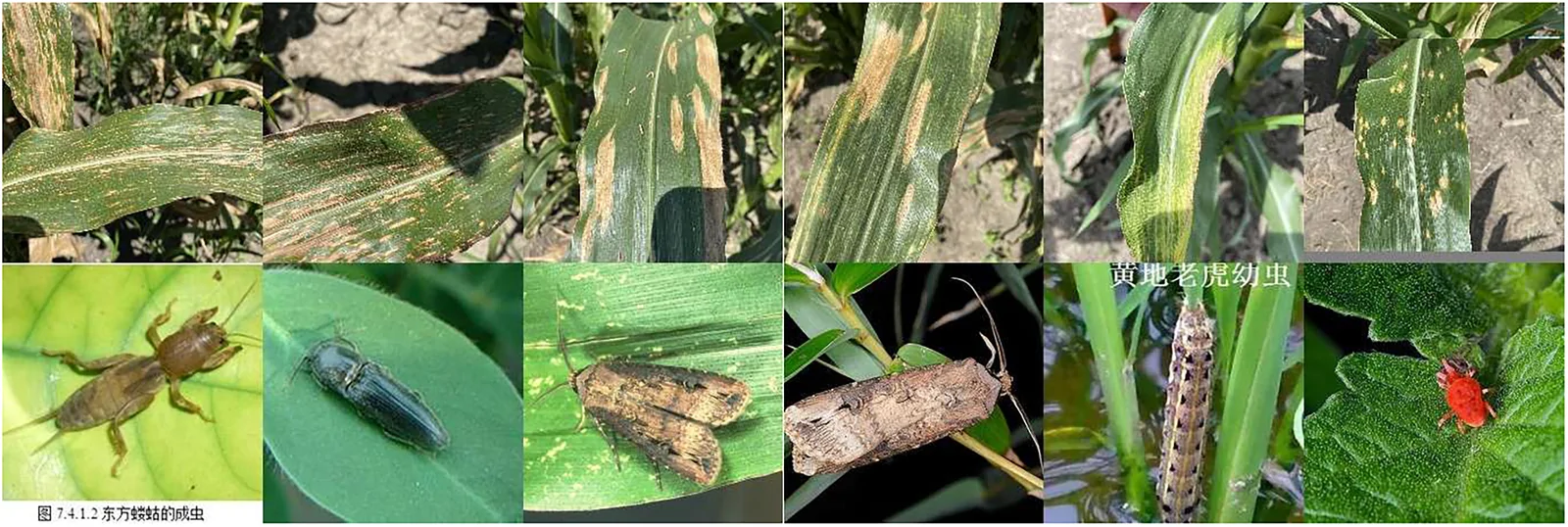
A few samples from both datasets, where the first row contains visuals from the CD&S dataset, while the second row indicates the visuals from the IP102.

To establish a reliable and unbiased evaluation protocol, both datasets were divided using a stratified image-level splitting strategy into training, validation, and testing subsets with a ratio of 70%, 10%, and 20%, respectively. Stratification was applied to preserve the class distribution across all subsets and minimize sampling bias, mostly for classes with limited samples. The partitioning was performed before model training, and each image was assigned exclusively to only one subset. Therefore, no image overlap existed between the training, validation, and testing sets to prevent potential data leakage. The validation and testing samples remained completely unseen during training to ensure an unbiased evaluation of the generalization capability of the proposed work. The experiments reported in this study were conducted using this predefined stratified partition to maintain a consistent evaluation protocol across all models. Although repeated runs with different random seeds and reporting mean ± standard deviation can provide further evidence of statistical stability, such repeated-run analysis is considered beyond the scope of the present experimental protocol and will be investigated in future work.

The training subset was used exclusively for optimizing the parameters of the PlantDefender framework, whereas the validation subset was used for monitoring convergence and selecting the optimal model configuration. Specifically, the model performance was evaluated on the validation set after each training epoch, and the checkpoint achieving the best validation performance was retained for final evaluation. The independent test subset was utilized only after completion of training and model selection to report the final results. This evaluation strategy ensures that the reported performance represents the actual generalization ability of the proposed framework.

Before training, all input images were resized to 299 × 299 pixels to match the input requirements of the modified InceptionResNet-V2 backbone and normalized using the standard preprocessing procedure. The proposed framework comprises approximately 59.4 million trainable parameters and requires approximately 26.4 GFLOPs per forward pass.No data augmentation techniques were employed in this study. This controlled setting was adopted to evaluate the feature-learning and detection capability of PlantDefender using the naturally occurring variations in the original datasets without introducing additional artificially transformed samples. Although augmentation strategies such as geometric transformations, scale variation, cropping, and illumination changes may potentially improve generalization, particularly for visually diverse pest instances in IP102, their systematic impact was beyond the scope of the present study. Evaluating augmentation strategies and their combinations represents an important direction for future work to further investigate their potential contribution to pest detection robustness.

The proposed PlantDefender framework was trained using the Adam optimizer with an initial learning rate of 0.0001, a batch size of 8, and 80 epochs. Adam was selected to provide stable optimization during fine-tuning of the refined InceptionResNet-V2 backbone and Faster R-CNN detection modules. The same preprocessing strategy, training configuration, and evaluation protocol were consistently applied across both datasets to ensure fair comparisons and reproducibility. All reported results were calculated exclusively on the independent test subsets. **Table 1** shows the dataset partitioning and experimental protocol. The corresponding training and validation loss convergence curves for both classification and regression are presented in **Figure 5**, demonstrating the optimization behavior of the proposed framework across the training process.

**Table 1:** Dataset partitioning and experimental protocol.

Dataset	Classes used	Splitting strategy	Training	Validation	Testing	Augmentation
IP102	13 maize pest classes	Stratified image-level split	70%	10%	20%	None
CD&S	3 maize disease classes	Stratified image-level split	70%	10%	20%	None

**Figure 5:**
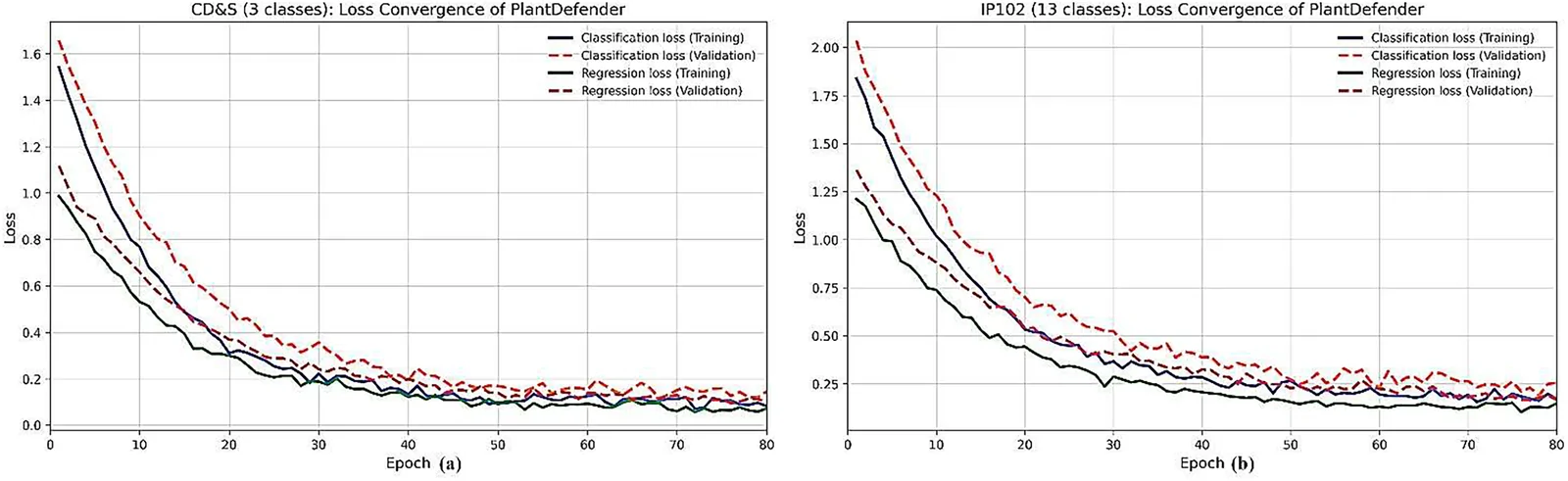
Loss convergance graph of the PlantDefender approach **(a)** CD&S dataset, **(b)** IP102 dataset respectively.

### 4.3 Model evaluation

This part represents the overall performance of the PlantDefender approach in recognizing the maize plant diseases and pests over the employed data samples.

First, we have discussed the results of the PlantDefender approach for pest classification employing the maize pest samples from the IP102 dataset. For this, we have computed the precision, recall, accuracy, and F1-score of the suggested approach and obtained values in the form of graphical representations provided in **Figure 6**. The values in **Figure 6** are clearly showing that our approach performed well in recalling various types of maize pests. Clearly, we have attained a precision of 78.12%, which indicates that the model accurately identifies plant pests with minimal false positives and ensures that most detected cases are true positives. This reliability is crucial for effective pest management and starting earlier interventions. Further, we have attained a recall of 79.06%, which proves that the proposed approach sustains a high recognition ability in distinguishing among the various types of maize plant pests. The F1-Score for our PlantDefender approach is 78.59% in recognition of the maize plant pests, which indicates that the model maintains a balanced performance between precision and recall and proves that it can effectively identify plant insects while minimizing both false positives and false negatives. This balance is essential for accurate and reliable pest detection. Finally, our accuracy score is 78.67%, signifying that the model correctly identifies pests in a large proportion of cases and proving the overall effectiveness of the approach in distinguishing between various categories of pests. Through the reported evaluation in terms of all standard metrics, we can say that the attained values on such a challenging dataset (IP102) with diverse environmental settings indicate the high recognition ability of our approach.

**Figure 6:**
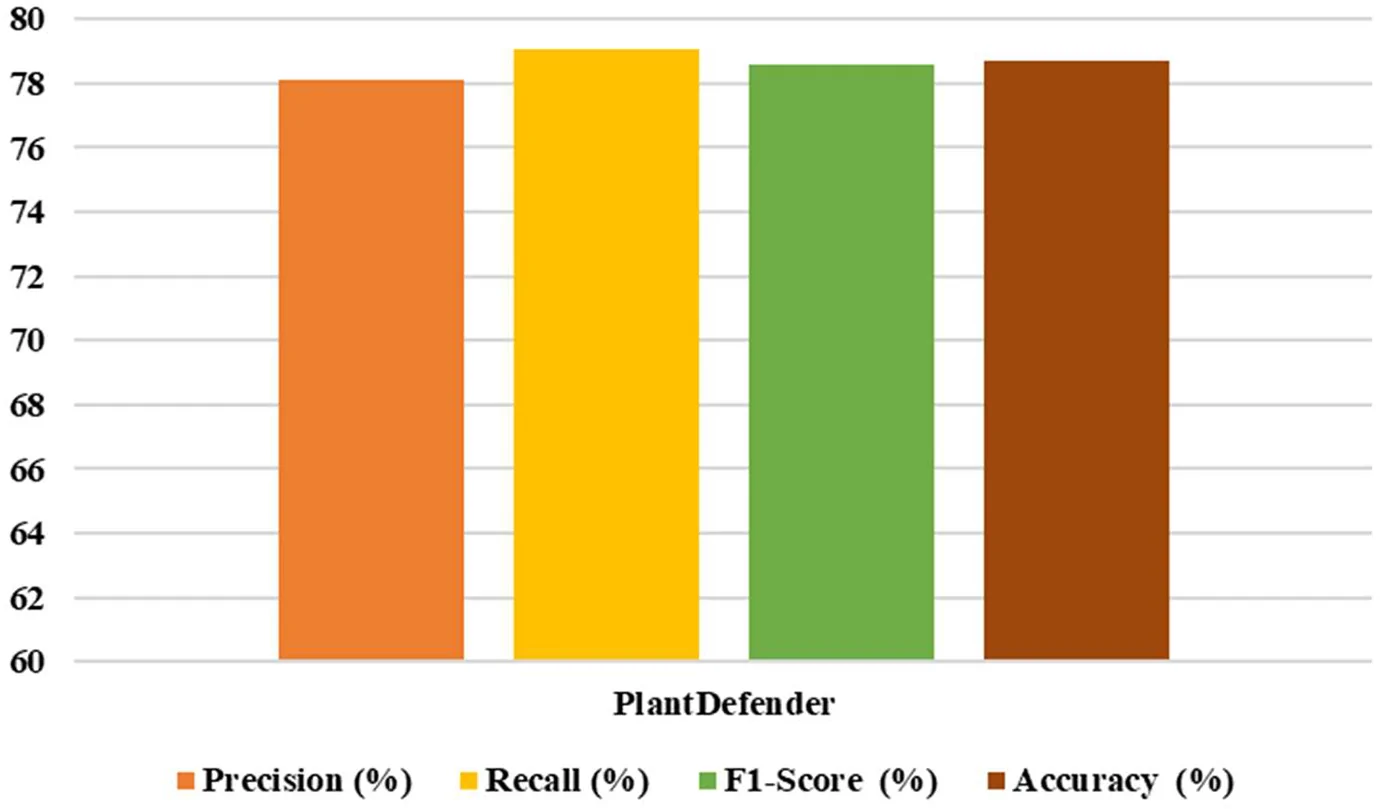
PlantDefender results attained on the pest samples from the IP102 dataset.

Next, we analyze the confusion matrix obtained by the PlantDefender approach for the 13 maize pest classes in the IP102 dataset. The confusion matrix, presented in **Figure 7**, provides a class-wise assessment of the model**’**s recognition performance and highlights the specific categories contributing to the overall error. The model achieves comparatively strong recognition across most pest categories; however, greater confusion is observed among the visually similar Black cutworm, Large cutworm, and Yellow cutworm classes. In particular, the class-wise recognition rates for Black cutworm, Large cutworm, and Yellow cutworm are 84.22%, 73.24%, and 74.39%, respectively. Reciprocal misclassification is evident among these classes: approximately 6.00% of Black cutworm instances are predicted as Large cutworm and another 6.00% as Yellow cutworm, while 6.00% of Large cutworm instances are classified as Black cutworm and 7.00% as Yellow cutworm. Similarly, 5.00% of Yellow cutworm instances are classified as Black cutworm and 7.00% as Large cutworm. This pattern indicates that the major source of error is complex inter-class similarity rather than a generalized inability to recognize pest instances. The similarity in morphology, body shape, color distribution, texture, and overall appearance makes it difficult to learn sufficiently discriminative features for these closely related categories. PlantDefender mitigates this challenge through the hierarchical and multi-scale feature representations learned by the refined InceptionResNet-V2 backbone, while the region-based Faster R-CNN architecture further exploits localized features through region proposal generation, ROI processing, localization, and classification. The Swish-based refinement facilitates nonlinear feature learning within the backbone, contributing to the extraction of more discriminative representations. However, the residual confusion among the cutworm categories demonstrates the inherent difficulty of complex pest recognition in IP102. These class-wise observations provide a deeper interpretation of the 78.67% overall accuracy and 0.7790 mAP, which shows that the comparatively moderate overall performance is primarily influenced by challenging visually similar pest categories and the complex visual characteristics of the dataset.

**Figure 7:**
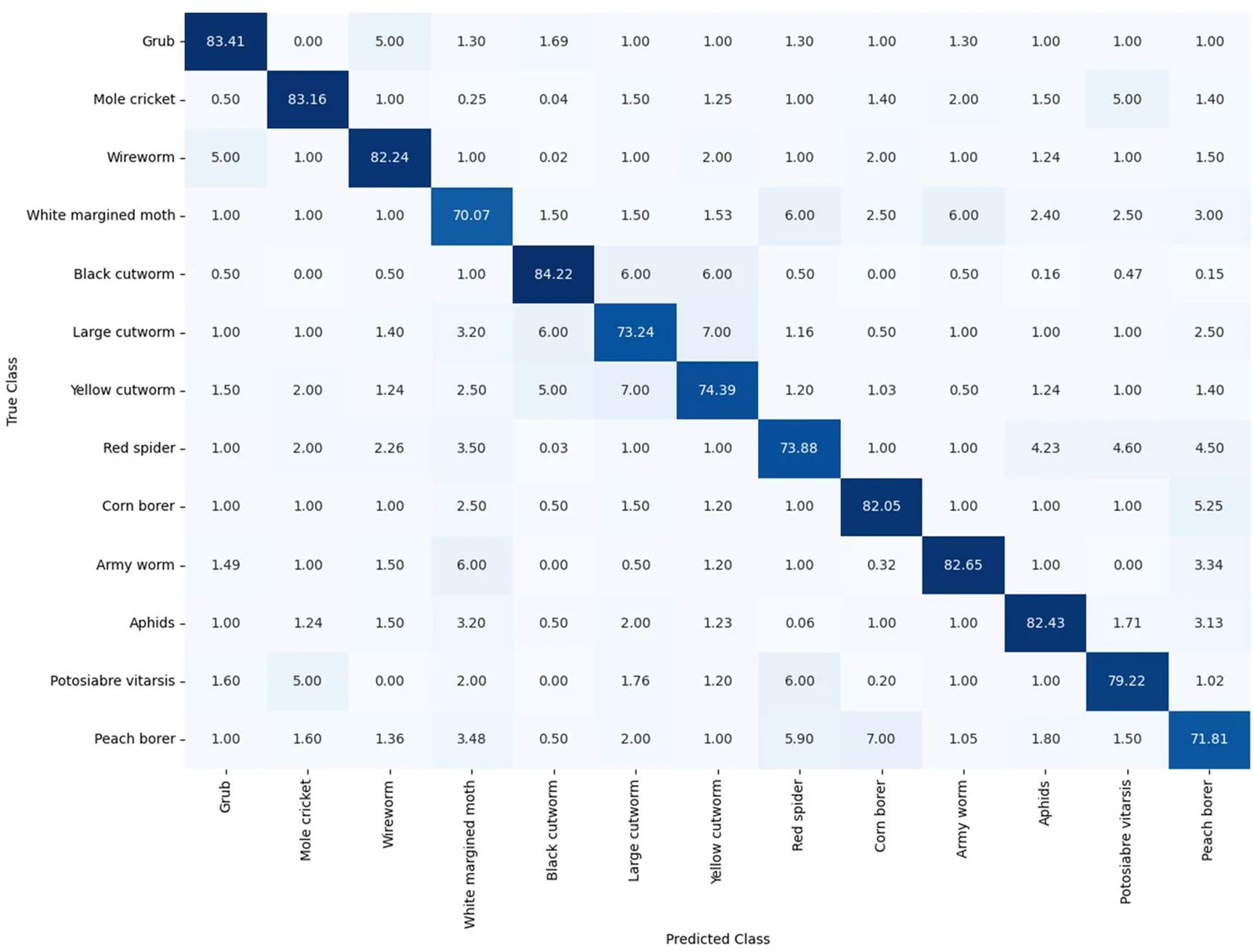
PlantDefender results in the form of a confusion matrix on the pest samples from the IP102 dataset.

Next, the results for the CD&S dataset have been discussed, attained by our approach, and performance values in terms of standard parameters are provided in **Figure 8**. Again, it confirms from all scores in **Figure 8** that the model performs well in classifying all classes of maize plant viruses. We have secured a precision of 98.51% over the employed dataset, which shows that the PlantDefender method has classified all classes effectively. Further, we have attained a recall of 98.83%, which indicates the high recall ability of our approach. The PlantDefender approach has attained an F1-Score and accuracy of 98.67%, and 98.44% that is proving the overall recognition ability of our approach.

**Figure 8:**
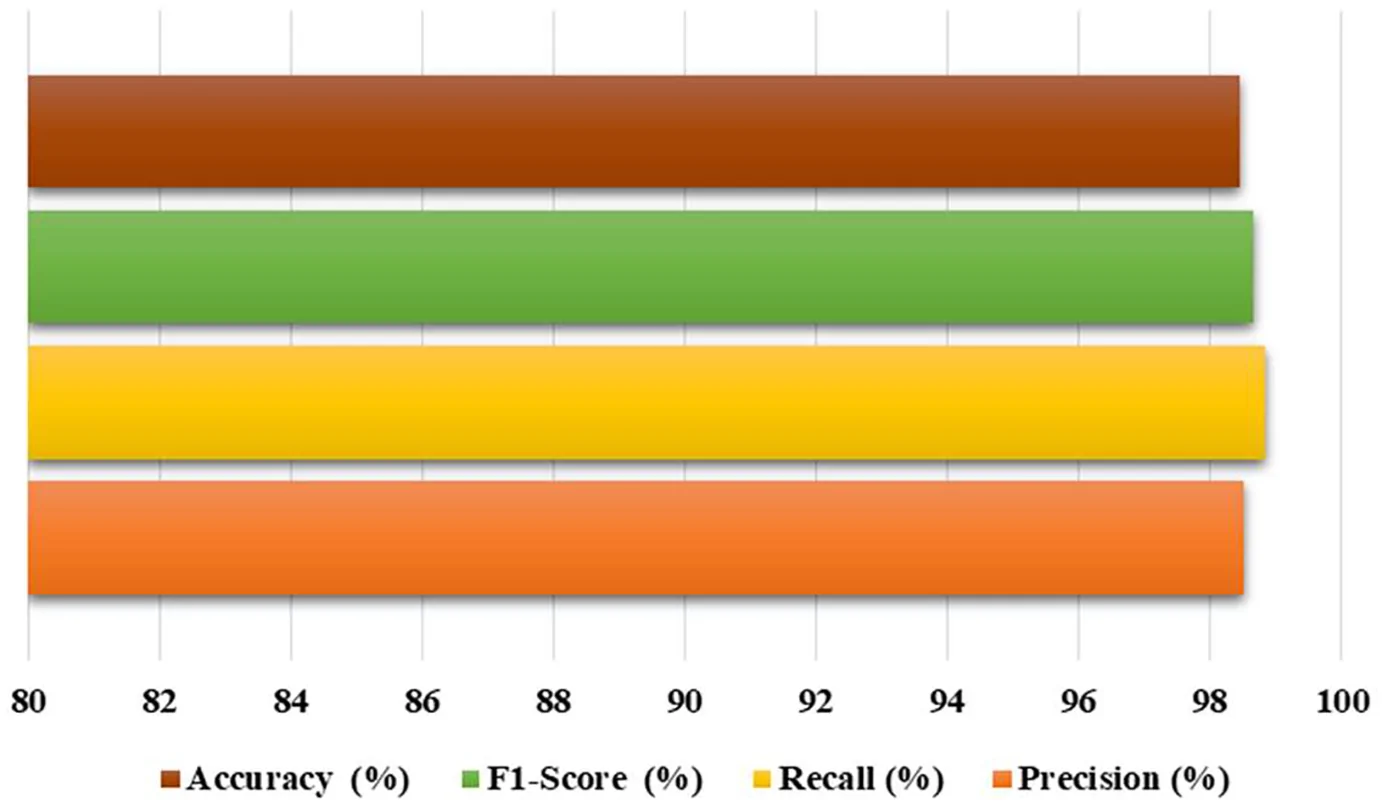
PlantDefender results attained on the maize plant disease samples from the CD&S dataset.

Next, we have discussed the confusion matrix attained by the PlantDefender approach for the CD&S data sample, as it provides a detailed review of the capability of the approach in classifying each individual class. The attained plot is given in **Figure 9**, which signifies that our solution is highly competent in recalling all groups of maize plant diseases. Further, it can be observed from the results that our work has obtained the highest TPR for the NLS group with a value of 99.61%, which shows that the approach is effective in recognizing this group of maize plant diseases. Further, it can be seen that the lowest TPR is reported for the GLS class with a score of 97.93%. This can be because of the reason that this group of maize plant diseases contains diverse symptom patterns, which causes the model to misclassify its signs. Further, the highest error rates are reported among the NLS and GLS classes, with scores of 0.95% and 1.78% due to the texture similarity of both groups. Overall, the PlantDefender approach performs well for all groups due to its high recall power, which can make it a powerful tool for the prompt intervention of such drastic maize plant diseases. Although the CD&S dataset provides a useful evaluation of maize disease detection, it contains only three disease categories and therefore does not represent the full diversity of diseases affecting maize. Consequently, the results obtained on CD&S should be interpreted within the scope of these three disease classes rather than as evidence of universal generalization to all maize diseases. Differences in disease appearance caused by cultivar, environmental conditions, geographic location, imaging devices, and disease severity may affect cross-domain performance. Future work will therefore investigate cross-dataset and external validation using additional maize disease datasets to more rigorously assess the generalization and domain-transfer capability of PlantDefender.

**Figure 9:**
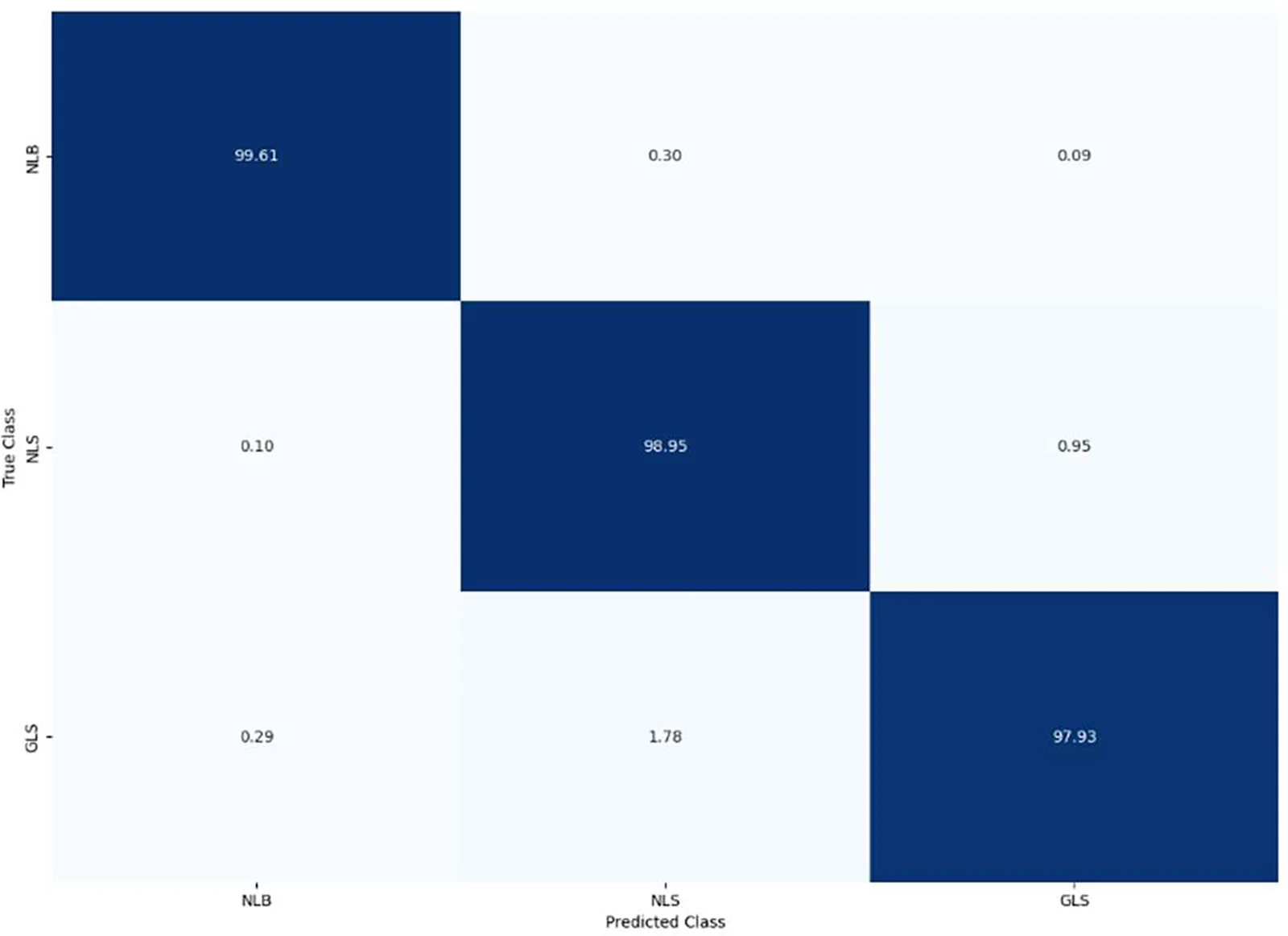
PlantDefender results in the form of a confusion matrix on the maize plant disease samples from the CD&S dataset.

After detailed evaluation on both datasets, PlantDefender demonstrates effective performance across the two considered maize health-detection scenarios. However, a substantial quantitative performance difference is observed between CD&S and IP102. On CD&S, the framework achieves an accuracy of 98.44% and an mAP of 0.9610, whereas on IP102 it achieves 78.67% accuracy and an mAP of 0.7790. Thus, the performance difference corresponds to 19.77% in accuracy and 0.1820 in mAP. In terms of classification error, the error rate is 1.56% on CD&S compared with 21.33% on IP102, representing a 19.77% increase for the pest detection task. This quantitative difference is consistent with the substantially different visual characteristics of the two datasets. CD&S primarily contains relatively stationary disease symptoms manifested through lesion patterns, discoloration, and texture variations on maize leaves, whereas IP102 contains insect instances exhibiting greater variation in appearance, scale, orientation, developmental stage, visibility, and background context. Moreover, the IP102 confusion matrix indicates that a considerable proportion of the errors is concentrated among visually similar pest categories, particularly Black cutworm, Large cutworm, and Yellow cutworm. Therefore, the lower IP102 performance is not interpreted simply as a general limitation of PlantDefender; rather, the quantitative performance gap and class-wise error patterns indicate that complex pest discrimination and greater visual variability constitute major challenges for the proposed framework.

### 4.4 Localization

As we have used an object detection approach in this work, which performs both the localization and classification of the targeted spots, therefore, this section comprises both the visual and numeric results of the PlantDefender approach in locating the infected plants and pests.

First, we have explained the recognition ability of the PlantDefender approach for locating the pests in the given images, and the attained visuals on the IP102 maize pest samples are provided in **Figure 10**. The samples clearly show that our approach is strong in locating pests of varying types and sizes in altered environmental settings. We have further assessed the localization power of the PlantDefender approach by calculating the mAP and IoU values due to their recognition as standard performance parameters in this domain. The PlantDefender approach has attained an average mAP of 0.7790 and an IoU score of 0.7531, which proves the high localization power of our approach on such a diverse and complicated pest sample.

**Figure 10:**
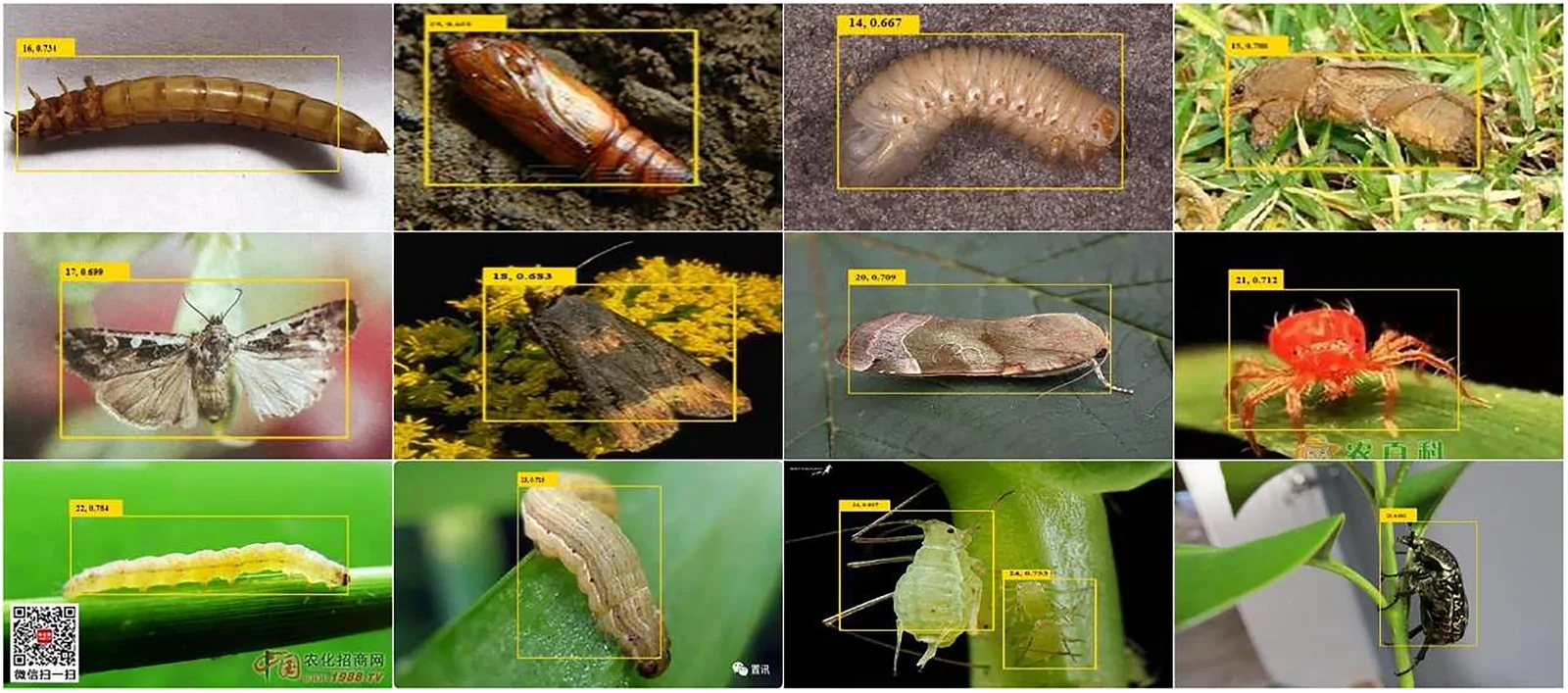
Localization results of the PlantDefender approach over the maize pests from the IP102 data sample.

Next, we have elaborated on the recognition ability of the PlantDefender approach for localizing the diseased maize samples. The attained visuals are given in **Figure 11**, which indicates that our solution again shows effective results in locating the diseased samples from all categories. In terms of numeric evaluation, we have attained an average mAP of 0.961 and an IoU of 0.9530, which signifies that the PlantDefender approach performs effectively in recognizing all categories of maize plant diseases with varying environmental conditions.

**Figure 11:**
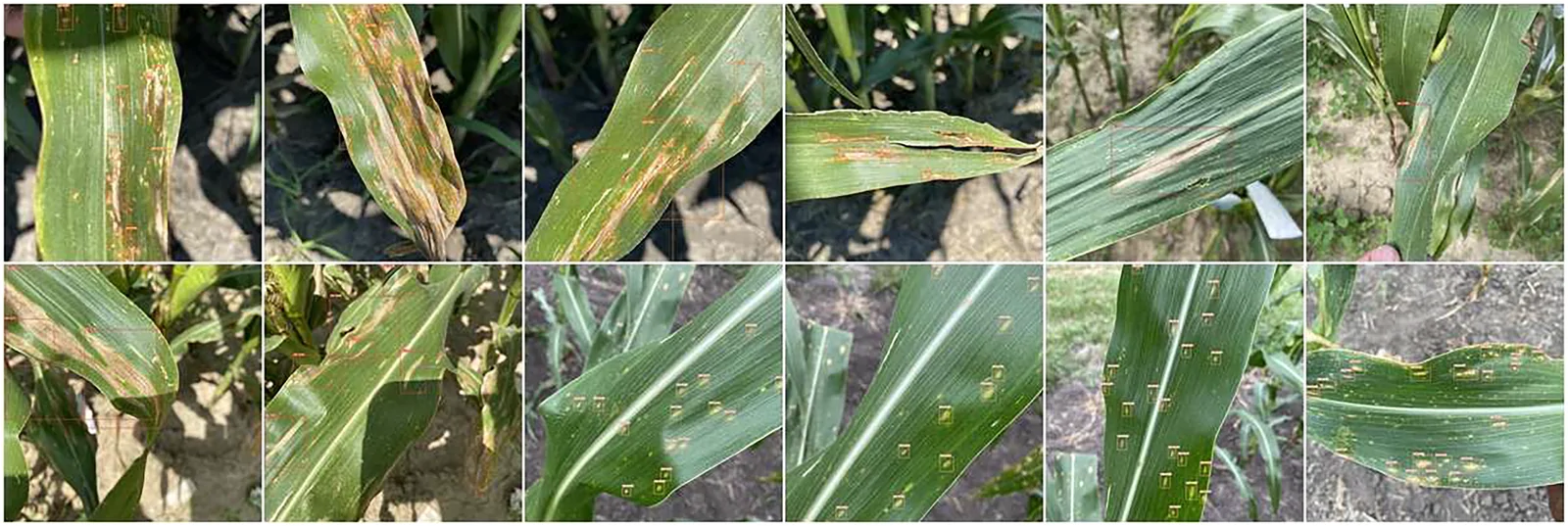
Localization results of the PlantDefender approach over the maize plant disease samples from the CD&S data sample.

Further, **Figure 12** presents representative failure cases in which the proposed model missed or incorrectly classified pest and disease instances. These errors can be attributed to the very small size of some targets, limited visual information, partial occlusion, variable illumination and shadows, background clutter, and substantial intra-class variation in pest appearance. In addition, visually similar disease symptoms and low-contrast targets can make it difficult to distinguish the affected regions from surrounding plant tissues.

**Figure 12:**
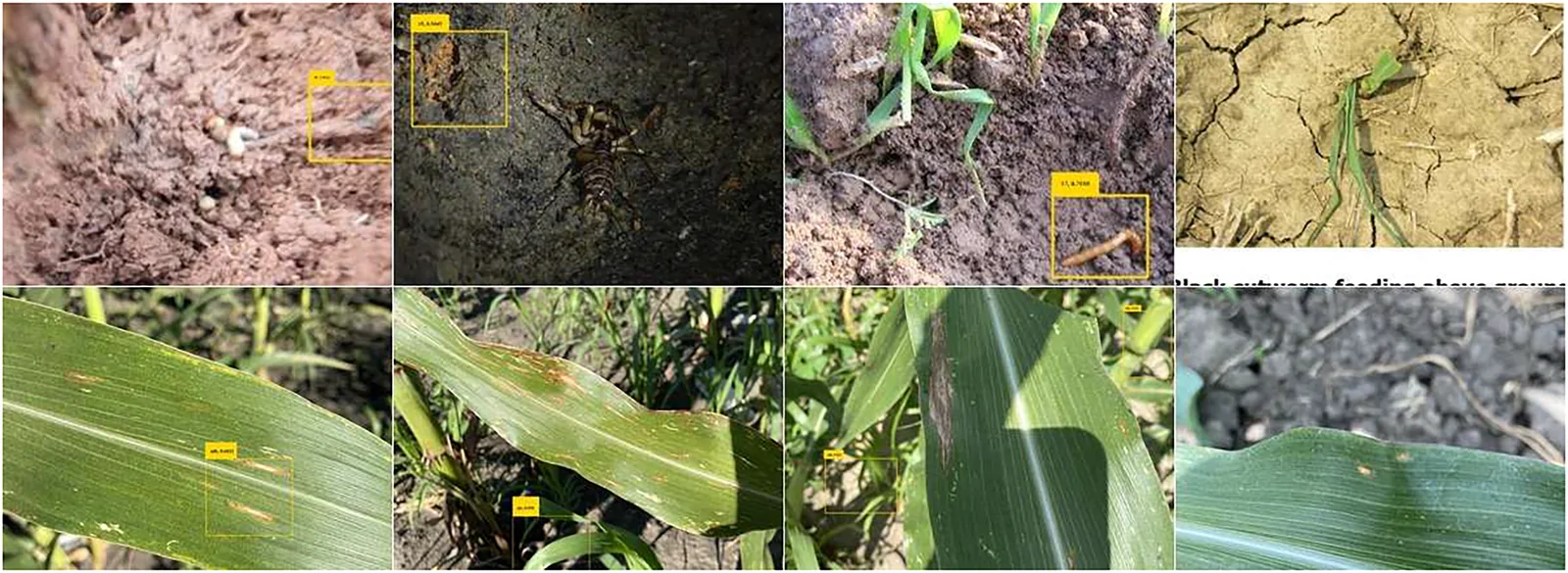
Sample visual results of false detection using the proposed approach.

Overall, the results obtained on both datasets demonstrate that the proposed approach is capable of not only recognizing plant diseases and pests but also accurately localizing their targeted regions. The improved results of the study are due to the incorporation of the refined feature extraction backbone of the Faster-RCNN model, which improves the information-capturing capability of the model and enhances its ability to tackle diverse sample conditions. The refined Faster-RCNN approach gets a more relevant set of sample information on which it successfully executes the localization and classification task. So, it can be said that the PlantDefender approach is more effective for both maize plant diseases and pest recognition.

### 4.5 Ablation study of activation functions

To investigate the contribution of the proposed activation-function modification, an ablation study was conducted by comparing the conventional ReLU-based InceptionResNet-V2 and the proposed Swish-based InceptionResNet-V2 within the same Faster R-CNN framework. All other architectural and experimental settings were kept unchanged to ensure that the observed differences were attributable to the activation function. As shown in **Table 2**, replacing ReLU with Swish improved the performance on both datasets. On IP102, the accuracy increased from 65.84% to 78.67%, while mAP improved from 0.6201 to 0.7790. Similarly, on CD&S, accuracy increased from 94.36% to 98.44%, accompanied by an increase in mAP from 0.8771 to 0.9610. Similar improvements were also observed in precision and recall across both datasets, further supporting the effectiveness of the Swish-based modification. These consistent improvements across both pest and disease detection tasks demonstrate that the Swish-based feature extractor provides more effective feature representations than the conventional ReLU-based configuration, supporting its inclusion in PlantDefender.

**Table 2:** Ablation study of ReLU and Swish activation functions within the faster R-CNN framework.

Model	Feature extractor	Dataset	Accuracy (%)	mAP	Precision (%)	Recall (%)
Faster R-CNN	ReLU-based InceptionResNet-V2	IP102	65.84	0.6201	66.28	66.51
		CD&S	94.36	0.8771	94.22	93.18
	Swish-based InceptionResNet-V2	IP102	78.67	0.7790	78.12	79.06
		CD&S	98.44	0.9610	98.51	98.83

### 4.6 Backbone ablation study

To further assess the effectiveness of the proposed feature extractor, a controlled backbone comparison was conducted by replacing the proposed refined InceptionResNet-V2 with commonly used Faster R-CNN backbones, namely VGG-16, ResNet-50, and ResNet-101. All configurations were evaluated under the same experimental settings to ensure a fair comparison. As presented in **Table 3**, the proposed configuration consistently achieved the highest classification accuracy on both datasets, obtaining 78.67% on IP102 and 98.44% on CD&S. On IP102, the proposed backbone also achieved the highest mAP of 0.7790, substantially outperforming VGG-16 (0.5841), ResNet-50 (0.6628), and ResNet-101 (0.6552). This improvement is due to the stronger multi-scale feature representation provided by InceptionResNet-V2 and the smoother nonlinear activation provided by Swish, which together facilitate the extraction of discriminative features from visually diverse pest instances. On CD&S, the proposed configuration achieved an mAP of 0.9610, which is higher than other backbones. The particularly substantial improvement on IP102 further suggests that the refined backbone is more effective for the challenging pest detection scenario involving small objects, substantial appearance variation, and complex backgrounds.

**Table 3:** Ablation study comparing different faster R-CNN feature extractors on IP102 and CD&S.

Model	Feature extractor	Dataset	Accuracy (%)	mAP
Faster-RCNN	VGG-16	IP102	59.34	0.5841
		CD&S	93.12	0.9113
	ResNet50	IP102	65.37	0.6628
		CD&S	95.63	0.9437
	ResNet-101	IP102	66.83	0.6552
		CD&S	96.18	0.9423
	Proposed	IP102	78.67	0.7790
		CD&S	98.44	0.9610

### 4.7 Assessment of the PlantDefender approach with DL methods

Since PlantDefender is an object-detection framework that performs both target localization and classification, its primary evaluation is based on detection-oriented metrics, including mAP, precision, recall, and F1-score. However, to further assess the image-level discriminative capability of the feature-extraction/classification component of the proposed framework, an additional classification-oriented experiment was conducted using several well-established DL architectures, including GoogleNet, AlexNet, VGG19, ResNet variants, EfficientNet-B0, InceptionResNet-V2, and ConvNeXt, as given in (Albattah et al., 2023; Masood et al., 2023). These architectures are primarily designed for image-level classification and do not inherently generate object bounding boxes; therefore, the accuracy values reported in **Table 4** are not interpreted as direct object-detection comparisons with PlantDefender. Rather, this experiment provides a controlled auxiliary assessment of the ability of the underlying learned representations to distinguish maize disease and pest categories at the image-classification level. This analysis is important because it complements the detection experiments by examining whether the proposed feature-learning strategy provides strong class-discriminative representations independent of the localization component. The detection-specific performance of PlantDefender is evaluated separately against object-detection approaches using appropriate localization and detection metrics. The classification accuracy values for this experiment are summarized in **Table 4**. PlantDefender achieves 98.44% accuracy on CD&S and 78.67% on IP102, while the other classification architectures exhibit lower reported classification accuracies. These results provide an indication of the discriminative capability of the proposed feature-learning strategy; however, they should not be interpreted as evidence that PlantDefender achieves superior object localization compared with these classification-only networks.

**Table 4:** Classification-oriented comparison of PlantDefender with DL methods on CD&S and IP102.

Model	CD&S accuracy(%)	IP102 accuracy(%)
GoogleNet	86.82	57.33
AlexNet	91.26	58.09
VGG19	92.51	58.89
ResNet50	95.78	66.64
ResNet101	93.51	65.90
EfficientNet-B0	96.74	68.51
InceptionResNetV2	92.05	66.33
ConvNeXt	93.45	64.48
Proposed	**98.44**	**78.67**

The bold values are presenting values attained by the proposed approach named PlantDefender.

The classification-oriented results demonstrate that the refined feature-extraction strategy used in PlantDefender can learn discriminative representations for maize disease and pest categories. PlantDefender achieves the highest classification accuracy among the evaluated architectures on both datasets, indicating its strong class-discriminative capability under the adopted experimental setting. However, the differences among these conventional classification architectures may arise from their varying feature-extraction capacities and architectural characteristics; therefore, these results are considered supporting evidence of recognition capability rather than a direct comparison of object-detection performance. In contrast, PlantDefender additionally incorporates the Region Proposal Network, ROI processing, bounding-box regression, and classification components of Faster R-CNN, enabling both localization and classification of maize diseases and pests. Accordingly, the detection capability of PlantDefender is evaluated separately in the subsequent experiments using detection-specific metrics.

### 4.8 Comparison of the PlantDefender approach with object detection models over the IP102 dataset

In this section, we have accomplished the performance comparison of the PlantDefender approach with object detection approaches that performed the recognition of plant pests using the IP102 dataset. We have performed this using various evaluation measures for several object locator approaches like RetinaNet (Wang et al., 2019), the VGG-16-based Faster-RCNN approach (Nawaz et al., 2021), and the YOLO-V7 model (Wang et al., 2022) as given in (Yang et al., 2023). The attained evaluation is given in **Table 5**, from which it can be seen that our approach performs better than all other nominated models. Clearly, the VGG16-based Faster-RCNN approach shows the minimum performance with the precision and mAP of 48.20% and 55%, respectively. While, the second-lowest values are attained by the RetinaNet approach with a mAP of 65.90%. The YOLO-V7 approach shows the second-lowest precision rate with a value of 69%, however, in terms of localization, this technique attains better outcomes with a mAP of 70.80%. Comparatively, our PlantDefender model performs for all performance measures with a precision of 78.12%, along with the mAP of 77.90%. More clearly, for precision the comparative object locator approaches have attained an average score of 63.13%, which is 78.12% for our model. Therefore, in the aspect of the precision metric, we have acquired a performance improvement of 14.99%. Further, for the recall and F1 scores, the competitor approaches have reported average scores of 62.51% and 59.43%, which is 79.06% and 78.59% for the suggested approach. So, for both mentioned evaluation measures, the PlantDefender approach has reported improvements of 16.55% and 19.16%. Similarly, for the mAP measure, we have attained an improvement of 14%. These result enhancements are because of the better information computation skills of our work. Models like RetinaNet face issues with detecting small pests in cluttered backgrounds due to their single-stage design, which results in lower accuracy. The VGG16-based Faster R-CNN faces limitations in its ability to detect pests due to its relatively shallow architecture, which makes it less effective for feature extraction for small and cluttered pest features. YOLO-v7 lacks the capability to recognize smaller pests and suffers from decreased accuracy in complex environments. Our PlantDefender approach overcomes these issues by utilizing an enhanced Faster-RCNN architecture with refined InceptionResNetV2 for superior feature extraction. The inclusion of Swish activation in the convolution layers of the feature extractor results in improved gradient flow and efficiency, which provides a highly relevant set of image information to the Region Proposal Network of the suggested model to accurately detect pests in varied and challenging conditions.

**Table 5:** PlantDefender approach analysis with object detection methods over the IP102 sample.

Model	Precision (%)	Recall (%)	F1-Score (%)	mAP (%)
RetinaNet	72.20	56.53	61.80	65.90
Faster-RCNN	48.20	59.90	46.50	55
YOLO-v7	69	71.10	70	70.80
Proposed	**78.12**	**79.06**	**78.59**	**77.90**

The bold values are presenting values attained by the proposed approach named PlantDefender.

### 4.9 Comparison of the PlantDefender approach with object detection over the CD&S dataset

Here, we have compared the maize plant leaf disease recognition ability of our PlantDefender approach with various object recognition frameworks that accomplished the same task using the CD&S dataset. To perform this, various object locator approaches like YOLO-V3 (Redmon and Farhadi, 2018), SSD (Liu et al., 2016), VGG-16-based Faster-RCNN (Nawaz et al., 2021), and ResNet50-based Faster-RCNN (Masood et al., 2023) are taken as given in (Masood et al., 2023). For performance considerations, we have taken into account both the localization ability and model efficiency using the mAP and time metrics, respectively, and the analysis is provided in **Table 6**. The scores in **Table 6** indicate the superiority of PlantDefender in both evaluation aspects. The results clearly indicate that the SSD approach shows the lowest recognition score of 0.71. Further, the second-lowest localization result is obtained by YOLO-V5 with a mAP of 0.87. The YOLO-V5 approach also attains the highest test time of 0.34 seconds. In terms of model efficiency, the YOLO-V3 approach performs better with a test time of 0.19 seconds; however, its recognition results are not satisfactory, with a score of 0.87. The Faster-RCNN approach performs better in terms of localization ability with a mAP of 0.94; however, the approach is not computationally efficient, showing a test time of 0.26 seconds per image. The proposed PlantDefender approach performed better than all approaches in terms of maize plant disease recognition, with mAP of 0.961, and presents a better trade-off for model efficiency with a test time of 0.22 seconds per image. The improved outcomes of our study are because of its aptitude to deal with the issues of other discussed models. The SSD model fails to fully capture the detailed information of the suspected samples because of its simple architectural description. The YOLO-V3 approach is computationally efficient but unable to locate the small-sized diseased portions, which degrades its localization ability. ResNet50 and VGG16-based Faster R-CNN models face challenges in recognizing maize plant leaf diseases due to their limited capacity to extract relevant details and vulnerability to overfitting in complex backgrounds. These models struggle with the huge sample variations and complex patterns present in maize leaf diseases. Our PlantDefender approach addresses these issues by incorporating a refined InceptionResNetv2, which utilizes Swish activation to enhance gradient flow and feature discrimination. This advanced architecture allows for more precise extraction of detailed features, which assists us in increasing the accuracy of disease recognition even in complex and diverse agricultural environments as compared to its base models. Further, the employment of the swish activation method improved the efficiency of the PlantDefender approach by accelerating convergence during training, which reduced the overall computation time and sped up the model efficiency in contrast to its peer models. So, we can say that PlantDefender demonstrates effective performance on the evaluated maize diseases.

**Table 6:** PlantDefender approach analysis with object detection methods over the CD&S dataset.

Model	mAP	Time(s)
YOLO-V3	0.87	0.19
SSD	0.71	0.34
VGG16-based Faster-RCNN	0.89	0.23
ResNet50-based Faster-RCNN	0.94	0.26
Proposed	**0.961**	**0.22**

The bold values are presenting values attained by the proposed approach named PlantDefender.

Further, it can be seen from **Table 6** that although PlantDefender achieved an inference time of 0.22s per image, practical deployment on mobile devices or drones can be influenced by hardware limitations such as memory, battery life, and computational power. The attained speed suggests feasibility for near real-time crop monitoring, particularly in periodic drone surveillance and mobile-assisted diagnosis. Further optimization through model pruning, quantization, and lightweight acceleration frameworks could improve deployment efficiency on edge platforms.

### 4.10 Comparison of the PlantDefender approach with SOTA methods over the IP102 dataset

In this part, we have executed the comparative analysis of the PlantDefender approach with new methods (Albattah et al., 2023; Yang et al., 2023) that attempted to perform pest recognition using the IP102 dataset. To conduct the analysis, various standard parameters, including accuracy, recall, F1, and precision, are taken, and the obtained comparison is given in **Table 7**. The results in **Table 7** clearly show that our approach has performed better than the other latest techniques. The work in (Albattah et al., 2023) employed an object detection approach to localize and classify the crop pests from the inspected samples. To do so, the approach utilized a refined CornerNet approach where the DenseNet-100 model was used to obtain the visual aspects of the images, which were later localized and categorized through the regressor and classifier units of the CornerNet approach. The work has reported an average accuracy score of 70.70% for the maize pest classes along with a precision of 55.10%. While the work in (Yang et al., 2023) employed the refined YOLO-V7 framework for the recognition of the maize plant pests and reported a precision score of 73.30% along with a recall of 77.30% and mAP of 76.30%. Comparatively, our approach outperformed other approaches in the aspect of all performance parameters and reported an accuracy of 78.67%, along with precision, recall, and mAP of 78.12%, and 79.06%, 77.90%. These results demonstrate that PlantDefender provides improved precision, recall, and mAP compared with the reported results of (Yang et al., 2023), indicating its competitive performance for maize pest recognition on the evaluated IP102 classes. The work in (Albattah et al., 2023) faced limitations in recognizing crop pests due to its inability to identify small and densely clustered pests, which leads to inaccuracies in detecting insects against complex agricultural backgrounds. Moreover, the CornerNet model highly relies on corner pooling and struggles with the diverse outlines and appearances of pests, which compromises its overall detection performance. While the work discussed in (Yang et al., 2023) utilized YOLOV7, which struggles to locate the small-sized plant pests and is unable to tackle distorted samples. In comparison, the suggested PlantDefender approach overcomes these issues by utilizing a refined InceptionResNetv2, which results in effective feature extraction. Such refined information enhances the ability of the approach to distinguish small pests within complex backgrounds. The improved Faster-RCNN technique provides improved localization and classification capabilities by minimizing false positives and improving accuracy in densely cluttered environments. So, based on the performed analysis, it canbe concluded that our approach is more effective than latest works which we prove through ensuring precise and reliable pest detection.

**Table 7:** PlantDefender approach analysis with SOTA methods over the IP102 dataset.

Reference	Precision(%)	Recall(%)	F1-score(%)	Accuracy(%)	mAP
(Albattah et al., 2023)	55.10	54.40	54.70	70.70	-
(Yang et al., 2023)	73.30	77.30	75.20	-	76.30
Proposed	**78.12**	**79.06**	**78.59**	**78.67**	**77.90**

The bold values are presenting values attained by the proposed approach named PlantDefender.

### 4.11 Comparison of the PlantDefender approach with SOTA methods over the CD&S dataset

In this part, we have performed a comparison of the latest works (Chen et al., 2022; Ahmad et al., 2023; Divyanth et al., 2023; Masood et al., 2023) with our approach, which performed the classification of maize plant leaf diseases using the CD&S dataset. The obtained evaluation is discussed in **Table 8** with the help of several performance evaluators to quantitatively compare all works. The reported analysis clearly stated that the PlantDefender approach has reached the highest scores as compared to all other works. Ahmad et al. (2023) discussed several deep learning models to perform the categorization of maize plant ailments and obtained the highest accuracy of 81.60% for the DenseNet-169 approach. The method in (Chen et al., 2022) first utilized the Simple Linear Iterative Clustering (SLIC) technique to identify the infected plant leaf regions. In the next step, several deep learning frameworks were used to compute the visual aspects of the clustered areas to perform the classification task. The model attained the highest classification accuracy of 97.77% by using the DenseNet-121 approach. Divyanth et al. (2023)utilized a clustering phase with the employment of different segmentation techniques like DeepLabv3+, UNet, etc to locate the focused areas on which the classification task was executed. The approach has attained a classification accuracy of 92.37%. Lastly, the technique found in (Masood et al., 2023) utilized the Faster-RCNN approach with a ResNet50 base for locating and classifying the diseased plant leaves. This work has attained a classification accuracy of 97.89%. Whereas the suggested PlantDefender approach has acquired the largest classification accuracy score of 98.44%, that is showing the effectiveness of our approach. The main cause for this improved classification score is that our method has tackled the issues of existing approaches. The work discussed in (Ahmad et al., 2023) is unable to exactly identify the diseased regions and lacks the capability of handling cluttered and multiple objects. Comparatively, our approach has resolved this issue by proposing a more effective feature computation backbone that enables it to both locate and classify the infected portions of leaves, even under diverse environmental settings, and is proficient in recognizing multiple occurrences of diseased regions in a single leaf. While the methods in (Chen et al., 2022; Divyanth et al., 2023) utilized an additional clustering phase to identify the infected portions, this increases the computing burden. Comparatively, our approach is independent of such extra steps and can accomplish both localization and classification through a single framework. Lastly, the work in (Masood et al., 2023) applied a ResNet50-based Faster-RCNN, which faced overfitting problems in complex agricultural environments and was unable to effectively compute the dense sample features under intense light changes. Our PlantDefender approach overcomes these issues by employing a refined InceptionResNetv2, which delivers effective feature extraction through Swish activation and ensures enhanced gradient flow and feature discrimination. This improvement allows for more accurate localization and grouping of maize plant viruses even in challenging conditions. So, based on the performed analysis, we can conclude that our proposed solution provides an effective strategy to learn a denser and more reliable set of sample information, which ultimately enhances its recognition results.

**Table 8:** PlantDefender approach analysis with SOTA methods over the CD&S dataset.

Reference	Accuracy(%)
(Ahmad et al., 2023)	81.60
(Chen et al., 2022)	97.77
(Divyanth et al., 2023)	92.37
(Masood et al., 2023)	97.89
Proposed	**98.44**

The bold values are presenting values attained by the proposed approach named PlantDefender.

## 5 Conclusion

The work is based on proposing a unified model called PlantDefender capable of recognizing both maize plant diseases and pests. We have presented an improved Faster-RCNN approach by using a refined InceptionResNet-V2 model as its base network. Descriptively, the conventional InceptionResNet-V2 model is modified by replacing the conventional ReLU activation with Swish to ensure better gradient flow through the network, mitigate the vanishing gradient problem, and improve the aptitude of the work to acquire complex features more effectively. The computed information is later processed through the RPN, ROI Align, regression, and classification modules of the PlantDefender approach to accurately identify and categorize diseases and pests in maize crops. For performance evaluation, 2 standard repositories are used: IP102 comprise pest species damaging to maize, and the CD&S sample comprises visuals of common maize plant diseases. We have confirmed the effectiveness of the PlantDefender method in contrast to traditional models by conducting an extensive experimental analysis. We have attained an accuracy of 78.67% for recognizing maize pests from the IP102 dataset, and 98.44% for maize plant diseases over the CD&S dataset. Future work will extend the evaluation to recently proposed YOLO variants under controlled settings to further validate the robustness of PlantDefender. Future evaluation on additional independent maize disease datasets with broader disease coverage will further assess the cross-dataset generalization of PlantDefender. Additionally, exploring the use of more advanced activation functions like Mish and feature extraction techniques could further enhance the accuracy and efficiency of the approach. PlantDefender can also demonstrate strong potential for transfer learning-based adaptation to other crops such as wheat, rice, and tomato. Since the refined InceptionResNet-V2 backbone captures generalized disease- and pest-related visual representations, the framework can potentially adapt to new agricultural domains through fine-tuning on limited annotated samples rather than complete retraining. However, adaptation performance may vary depending on the similarity of disease symptoms and pest characteristics across crop species.

## Data Availability

The original contributions presented in the study are included in the article/supplementary material. Further inquiries can be directed to the corresponding authors.
